# SIRT5-mediated BCAT1 desuccinylation and stabilization leads to ferroptosis insensitivity and promotes cell proliferation in glioma

**DOI:** 10.1038/s41419-025-07626-9

**Published:** 2025-04-07

**Authors:** Tao Wang, Xin-Hao Han, Jun-Jun Chen, Xing Wang, Zhen Zhang, Xiao-Jian Han, Zhuo Lu

**Affiliations:** 1 Institute of Geriatrics, Jiangxi Provincial People’s Hospital, The First Affiliated Hospital of Nanchang Medical College, Nanchang, Jiangxi China; 2 Centre for Medical Research and Translation, Jiangxi Provincial People’s Hospital, The First Affiliated Hospital of Nanchang Medical College, Nanchang, China; 3 Institute of Clinical Medicine, Jiangxi Provincial People’s Hospital, The First Affiliated Hospital of Nanchang Medical College, Nanchang, China; 4 Department of Thoracic Surgery, The First Affiliated Hospital, Jiangxi Medical College, Nanchang University, Nanchang, Jiangxi China; 5 Jiangxi Hospital of China-Japan Friendship Hospital, National Regional Center for Respiratory Medicine Nanchang, Nanchang, Jiangxi China

**Keywords:** CNS cancer, Ubiquitylated proteins, Acetyltransferases, Cell death

## Abstract

Glioma is a highly aggressive brain tumor with limited treatment success due to its resistance to conventional therapies. Sirtuin 5 (SIRT5) has emerged as a promising target for cancer therapy, though it exhibits dual roles in different cancer types. In this study, we investigate the role of SIRT5 in glioma and its corresponding mechanisms. Our findings demonstrate that SIRT5 expression is elevated in glioma cells both in vitro and in vivo. SIRT5 knockdown significantly reduced glioma cell proliferation and enhanced sensitivity to ferroptosis. Proteomic and metabolomic analyses identifies branched-chain amino acid (BCAA) metabolism as a key downstream pathway regulated by SIRT5 through branched-chain aminotransferase 1 (BCAT1). Specifically, SIRT5-mediated desuccinylation of BCAT1 at K39 inhibits its interaction with the E3 ligase CHIP, thereby preventing BCAT1 degradation via the ubiquitin-proteasome system. Moreover, BCAT1 overexpression reverses the proliferation inhibition and ferroptosis sensitivity observed in SIRT5-knockdown cells. Clinically, we reveal a positive correlation between SIRT5 and BCAT1 levels in glioma samples, with higher expression levels predicting more advanced glioma grades and poorer clinical outcomes. Collectively, this study highlights the critical role of SIRT5 in promoting glioma progression via metabolic regulation and ferroptosis insensitivity, offering a potential therapeutic target for glioma treatment.

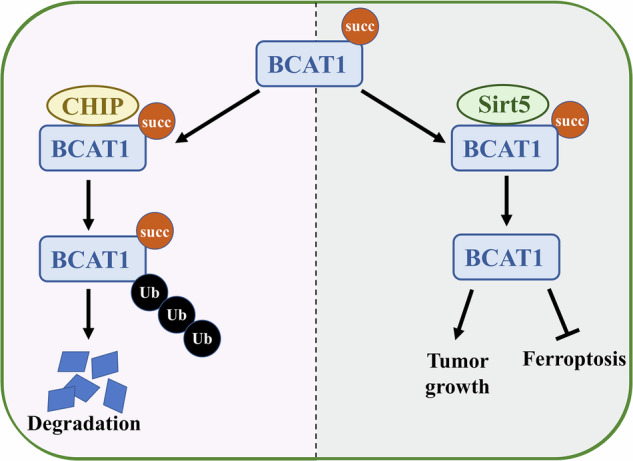

## Introduction

Gliomas, particularly glioblastomas (GBM), are among the most aggressive and lethal forms of primary brain tumors. Despite advancements in surgical methods, radiotherapy, and chemotherapy, patient outcomes remain poor, with frequent recurrences and a median survival of less than 15 months [1]. This dismal outcome is largely attributed to the invasive nature of glioma cells, their resistance to conventional therapies, and their ability to avoid programmed cell death mechanisms, including ferroptosis [2]. Recent research has increasingly concentrated on the metabolic alterations of glioma cells, particularly in glutamine metabolism and lipid metabolism, highlighting its significant role in cancer progression and chemoresistance [3]. However, the molecular basis of these processes, especially the regulation of metabolic pathways and their link to therapeutic resistance, is still not well understood.

One promising research avenue involves sirtuins, a family of NAD^+^-dependent deacylases that are involved in various biological processes, including aging, metabolism and stress response [4]. Of the seven members in the sirtuin family (SIRT1–SIRT7), SIRT5 has recently garnered attention for its role in controlling metabolic pathways [5], mitochondrial function [6], and oxidative stress [7]. Unlike other Sirtuins primarily noted for their deacetylase activity, SIRT5 exhibits unique desuccinylase activity, highlighting its distinct role in post-translational modifications [8]. In recent years, growing evidence has linked SIRT5 to cancer, suggesting that its function can either promote or suppress tumors, depending on the specific tissue and context [9]. For instance, SIRT5 desuccinylates S100 calcium-binding protein A10, promoting its degradation and thereby suppressing gastric cancer metastasis [10]. Lower SIRT5 expression has been observed in pancreatic ductal adenocarcinoma, where SIRT5 deacetylates glutamate oxaloacetate transaminase 1, hindering glutamine, glutathione, and pyrimidine metabolism, ultimately suppressing tumor growth [11]. However, in colorectal cancer, SIRT5 promotes cell proliferation and maintains redox balance by desuccinylating the K280 residue of the mitochondrial enzyme serine hydroxymethyltransferase [12]. Additionally, in breast cancer, SIRT5 has been shown to inhibit autophagy and mitophagy via regulating glutamine metabolism, which aids tumor cells in withstanding chemotherapy [13]. Despite these advances, the specific role of SIRT5 in glioma, particularly in relation to therapeutic resistance and metabolic regulation, has not been fully elucidated, representing a critical gap in the current literature.

Ferroptosis, characterized by the accumulation of lipid peroxides, is an iron-dependent form of cell death, distinguishing it from necrosis and apoptosis [14]. Iron catalyzes reactive oxygen species (ROS) production through the Fenton reaction, triggering lipid peroxidation of polyunsaturated fatty acids, leading to membrane damage and cellular disintegration [15]. GPX4, supported by GSH, is pivotal in counteracting ferroptosis by neutralizing lipid peroxides. GSH is synthesized from glutamate, which can be derived from glutamine and α-ketoglutarate [16]. Ferroptosis-inducing agents like Erastin and RSL3 either inhibit cystine import or deactivate GPX4 directly, resulting in GSH depletion and subsequent cell death through lipid peroxide buildup [17]. Previous research connects ferroptosis resistance with glioma development and poor prognosis [18, 19]. Sulfasalazine (SAS), an FDA-approved anti-rheumatic drug, has been explored for its antitumor effect in glioma through ferroptosis induction [20, 21]. Nonetheless, elevated GPX4 and SLC7A11 levels, along with reduced ferroptosis markers, such as 12-HETE and 15-HETE, have been observed in glioma tissues and cell lines [18], suggesting glioma’s relative insensitivity to ferroptosis.

In this study, we investigate the expression, clinical relevance, and underlying mechanisms of SIRT5 in glioma. Our findings reveal high SIRT5 expression in glioma both in vitro and in vivo. SIRT5 knockdown suppresses glioma cell proliferation, confirming its role as oncogene. Furthermore, SIRT5 is shown to confer resistance to SAS-induced ferroptosis via the GSH/GPX4 axis. Additionally, we identify BCAT1 as a novel substrate for SIRT5-mediated desuccinylation. SIRT5 directly binds to BCAT1 and desuccinylates it at K39, which prevents CHIP-mediated ubiquitination and subsequent degradation of BCAT1. Overexpression of BCAT1 rescues the proliferation and ferroptosis resistance deficits caused by SIRT5 knockdown. Additionally, the expression of SIRT5 and BCAT1 is positively correlated with advanced glioma grade and unfavorable prognosis. Taken together, these findings suggest that SIRT5 plays a crucial oncogenic role in glioma and positions SIRT5 as a potential therapeutic target for ferroptosis induction to block tumor growth.

## Materials and Methods

### Cell culture

Human malignant glioblastoma multiforme U251 and U87 cell lines were grown in high-glucose Dulbecco’s Modified Eagle Medium (DMEM, SolarBio, #11995) containing 4 mM l-glutamine, and supplemented with 10% fetal bovine serum (FBS). Additionally, human microglial HMC3 cells were cultured in low-glucose DMEM supplemented with 10% FBS. The cells were maintained in an environment with 5% CO_2_ at 37 °C and a controlled humidity level. These cell lines were purchased from the National Collection of Authenticated Cell Cultures of the Chinese Academy of Sciences in Shanghai, China. Authentication of the cell lines was confirmed through short tandem repeat (STR) analysis performed by Biowing Applied Biotechnology (Shanghai, China). Furthermore, all cell lines were verified to be free of mycoplasma contamination.

### Cell transfection

SIRT5 siRNAs (Origene, #SR323578) were transfected into HMC3 and glioma cells using siTran 2.0 siRNA transfection reagent (Origene, #TT320001) according to the manufacturer’s instructions. Plasmids were transfected using MegaTran 2.0 plasmid DNA transfection reagent (Origene, #TT210003), respectively.

### Cell proliferation assay

In the Crystal violet assay, cells were initially seeded into a 24-well plate (NEST Biotechnology, Wuxi, China; #702001) and subjected to transfection with the specified plasmids or siRNAs, or treated with various concentrations of MC3482 (MedChemExpress, #HY-112587). Afterward, fixation was carried out using 4% paraformaldehyde, followed by staining with 0.1% crystal violet solution. To assess absorbance, the stain was dissolved in 10% acetic acid, and measurements were taken at a wavelength of 595 nm.

For the colony formation assay, cells were seeded into 12-well plates (NEST Biotechnology, #712001) and subjected to transfection with the specified plasmids or siRNAs. Post-transfection, cells were incubated for a period of 12–14 days, fixed with 4% paraformaldehyde, and stained with 0.1% crystal violet solution. Photographs of the stained wells were taken, and colony numbers were quantified.

In the EdU staining assay, cells were cultured on glass coverslips placed in 12-well plates. EdU staining was carried out using the EdU Imaging Kit (APExBio, Shanghai, China; #K1075) following the manufacturer’s guidelines. The stained cells were visualized with an inverted fluorescence microscope (Nikon Eclipse Ti2-U, Japan).

### Cell Counting Kit-8 (CCK8) assay

Cells were plated in 96-well plates (NEST Biotechnology, #701002) at a density of 5 × 10³ cells per well. Following the specified treatment, 10 µL of the Cell Counting Kit-8 reagent (AbMole BioScience, USA) was added to each well, and the plate was incubated at 37 °C. Absorbance at 450 nm was measured using a microplate reader (PerkinElmer EnSpire, Shanghai, China).

### Proteomic and metabolomic analysis

Proteomic analysis was carried out using tandem mass tag (TMT) mass spectrometry, provided by Wuhan IGeneBook Biotechnology Co., Ltd (Wuhan, China). The raw mass spectrometry data from each sample were processed using the MASCOT engine (Matrix Science, London, UK; version 2.2) within Proteome Discoverer 1.4 software for both identification and quantification of proteins. Differentially expressed protein sequences were further analyzed using the InterProScan and NCBI BLAST+ client software to identify homologous sequences. Gene ontology (GO) terms were assigned, and sequences were annotated using the Blast2GO software. Following this, the studied proteins were mapped to pathways by referencing the Kyoto Encyclopedia of Genes and Genomes (KEGG) database using KEGG orthology identifiers.

For metabolomic analysis, the samples were examined using an LC-ESI-MS/MS system (MS QTRAP® System from SCIEX and UPLC ExionLC AD), conducted by Wuhan IGeneBook Biotechnology Co., LTD The significant metabolites were identified by considering both a VIP score (VIP > 1) and a *P* value of less than 0.05 (Student’s *t*-test). VIP values, along with score and permutation plots, were extracted from the OPLS-DA results generated using the R package MetaboAnalystR. Data were log-transformed (log2) and mean-centered prior to OPLS-DA. A permutation test of 200 iterations was conducted to prevent overfitting. Identified metabolites were annotated by referencing the KEGG Compound database.

### Immunoprecipitation (IP), GST-pull down, and western blot assay

In the GST-pull down assay, recombinant SIRT5 protein (MedChemExpress, Shanghai China; #HY-P74545) was incubated with GSTSep Glutathione Magbeads (Yeasen Biotechnology, #20562ES03) along with GST-BCAT1 (Proteintech, #Ag4574) or GST (MedChemExpress, #HY-P70270) protein for 2 h at 4 °C. After incubation, the beads were washed three times with PBS and then boiled in 1×loading buffer for 10 min at 100 °C.

For the IP assay, cells were lysed in NP-40 lysis buffer, and the lysate was centrifuged at 12,000 × *g* for 20 min at 4 °C. The co-immunoprecipitation mixture, containing 30 μL of protein A/G agarose beads and 1 μg antibody, was incubated on a Rotator Shaker for 8 h at 4 °C. After incubation, the beads were washed three times with lysis buffer and boiled in 1×loading buffer for 10 min at 100 °C.

For Western blot analysis, proteins were separated using sodium dodecyl sulfate-polyacrylamide gel electrophoresis (SDS-PAGE) and then transferred to a polyvinylidene difluoride (PVDF) membrane. Membranes were blocked with 5% fat-free milk and incubated overnight at 4 °C with the following primary antibodies: SIRT5 (Proteintech, Wuhan, China; #15122-1-AP), BCAT1 (Proteintech, #13640-1-AP), HA (Proteintech, #51064-2-AP), Flag (Proteintech, #66008-4-lg), His (Proteintech, #66005-1-Ig), ubiquitin (Proteintech, #10201-2-AP), GPX4 (Cell Signaling Technology, #52455S), FTH1 (Proteintech, #83428-1-RR), and GAPDH (Proteintech, #10494-1-AP). The membranes were washed three times with Tris-buffered saline containing 0.1% Tween-20 before being incubated with HRP-conjugated secondary antibodies. Protein bands were visualized using a chemiluminescence detection kit, and images were captured with a Bio-Rad ChemiDoc MP digital gel image analysis system.

### Quantitative reverse transcription polymerase chain reaction (qPCR)

Total RNA was isolated using TRIzol reagent and subsequently reverse transcribed into complementary DNA (cDNA) using the M5 Sprint qPCR RT kit with gDNA remover (Mei5bio, Beijing, China; #MF949-01). Quantitative real-time PCR was carried out using M5 Hiper Realtime Super Mix with High Rox (Mei5bio, #MF013-505). The sequences for the primers used in the analysis are provided in Table S1.

### Immunohistochemistry (IHC) staining

Tumor microarrays were purchased from Shanghai Outdo Biotech Co., LTD. Sections of brain tissues (frontal lobe) were bought from Shanghai WeiaoBio Co., LTD. IHC staining of tumor microarrays, brain tissues, and xenografted tumors was carried out by Wuhan ServiceBio Technology Co., Ltd.

### Immunofluorescence assay

To detect the subcellular localization of SIRT5 and BCAT1, cells were fixed with 4% paraformaldehyde for at room temperature 20 min and permeabilized with phosphate-buffered saline (PBS) containing 0.5% Triton-X. Cells were then blocked with 5% goat serum and incubated at 4 °C overnight with the primary antibodies. On the Next day, they were washed three times with PBS containing 0.5% Tween-20, followed by incubation with fluorescent secondary antibodies (Proteintech, #RGAM002, #RGAR004). Nucleus was stained with 4′,6-diamidino-2-phenylindole (DAPI). Images were captured using an inverted fluorescence microscope (Nikon Eclipse Ti2-U).

To assess reactive oxygen species (ROS) levels, cells were incubated with 5 μM Dihydroethidium (DHE; MedChemExpress, #HY-D0079) for 30 min in the dark at 37 °C, then washed twice with PBS. To visualize oxidized lipids, cells were incubated with 10 μM BODIPY 581/591 C11(MedChemExpress, #HY-D1301) for 30 min in the dark at room temperature, then washed twice with PBS. Fluorescent images were taken using an inverted fluorescence microscope (Nikon Eclipse Ti2-U), and the fluorescence intensity was quantified through flow cytometry (BD FACS Canto® II).

### Measurement of intracellular glutathione (GSH), glutamate, lipid peroxidation (LPO), and malondialdehyde (MDA) levels

The levels of intracellular GSH, glutamate, LPO, and MDA were quantified utilizing the Reduced Glutathione Content Assay Kit (Solarbio, Beijing, China; #BC1175), Glutamic Acid (Glu) Colorimetric Assay Kit (Elabscience, #E-BC-K903-M), the Lipid Peroxide Content Assay Kit (Solarbio, #BC5245), and the Malondialdehyde Content Assay Kit (Solarbio, #BC0025), following the protocols outlined by the manufacturer.

### Stable cell line construction

The full-length BCAT1 sequence was inserted into pLV-EF1α-Bsd plasmid. HEK-293 cells were co-transfected with the recombinant plasmid along with pMD2.G and psPAX2 plasmids for lentivirus packaging. SIRT5 shRNA lentiviral stocks were purchased from Shanghai Genechem Co., Ltd (#GXDL0423531_1). U251 and U87 cells were then infected with the lentiviral stocks and selected using blasticidin S or puromycin, respectively. Primer sequences are detailed in Table S1.

### Xenograft model

The study was conducted under the approval of the Ethics Committee of Jiangxi Provincial People’s Hospital (protocol code KT078). Four-week-old male NOD/SCID mice were obtained from SPF Biotechnology Co. Ltd (Beijing, China) and were randomly assigned to groups. Each group of mice was housed in a Specific Pathogen-Free (SPF) animal facility and subcutaneously injected with 3 × 10⁶ cells. SAS (30 mg/kg) was administered daily via intraperitoneal injection. At the designated time points, the mice were sacrificed, and the xenografted tumors were collected. The maximal tumor size did not exceed 150 mm. The researcher was blinded to the group allocations throughout the experiment.

### Statistical analysis

Statistical significance between groups was determined using either a Student’s *t*-test or one-way analysis of variance (ANOVA), followed by Fisher’s LSD test. All data are presented as mean ± standard deviation (SD). A *p* value of less than 0.05 was considered statistically significant.

## Results

### SIRT5 expression is upregulated in glioma and plays a significant role in cell proliferation

**Fig. 1:**
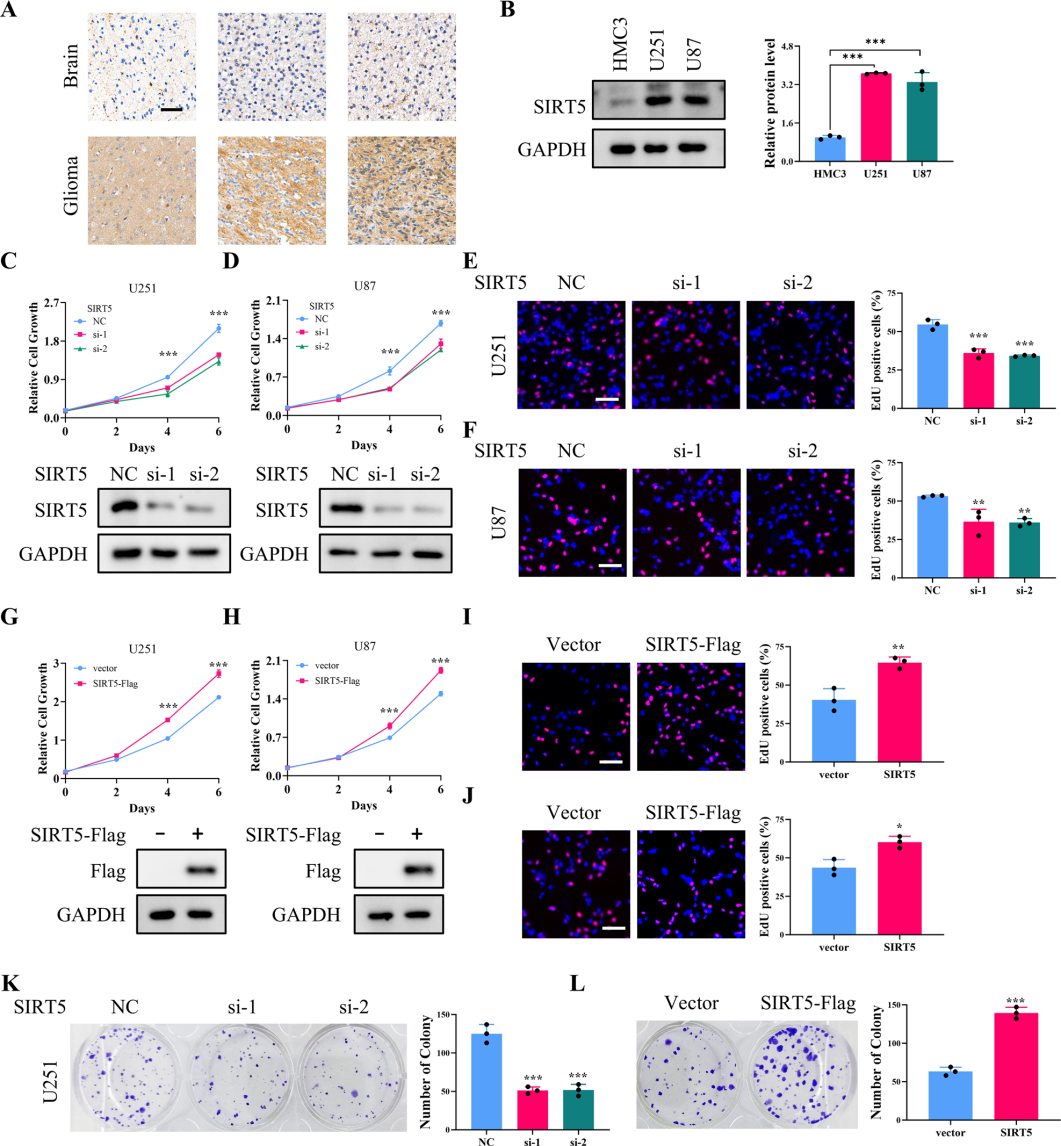
SIRT5 expression is upregulated in glioma and promotes cell proliferation. **A** SIRT5 expression in glioma tissues and normal brain tissues was determined by immunohistochemistry staining. Scale bar = 50 μm. **B** Western blot analysis of SIRT5 expression in HMC3, U251, and U87 cells. **C**–**J** Crystal violet staining and EdU staining were performed to evaluate the effect of SIRT5 knockdown or overexpression on the proliferative ability of U251 and U87 cells. Scale bar = 100 μm. **K**–**L** Colony formation assay was performed to evaluate the effect of SIRT5 knockdown or overexpression on the colony formation ability of U251 cells. Data are presented as mean ± SD of three independent experiments. *p < 0.01, **p < 0.01, ***p < 0.001.

In vivo immunohistochemistry staining revealed that glioma tissues exhibit higher levels of SIRT5 compared to normal brain tissues (Fig. 1A). This finding was further corroborated by western blot analysis, which demonstrated increased SIRT5 expression in glioma cell lines, U251 and U87, relative to HMC3 (Fig. 1B). To assess whether SIRT5 influences cellular proliferation, crystal violet and EdU staining were employed. Knockdown of SIRT5 resulted in a marked reduction in the proliferation of U251 and U87 cells (Fig. 1C–F), whereas overexpression of SIRT5 significantly accelerated their growth (Fig. 1G–J). Moreover, the knockdown of SIRT5 impaired the colony formation capability of U251 cells, while its overexpression had the opposite outcome (Fig. 1K–L). Additionally, treatment with MC3482, a specific SIRT5 inhibitor, also inhibited the proliferation of U251 and U87 cells (Fig. S1A, B). On the Contrary, SIRT5 knockdown had mild inhibition on HMC3 cell proliferation, while SIRT5 overexpression exerted no effect, indicating that SIRT5 plays a tumor-specific role in glioma cells (Fig. S1C, D). Collectively, these findings suggest that SIRT5 upregulation plays a pivotal oncogenic role in glioma progression.

### SIRT5 knockdown enhances the sensitivity of glioma cells to ferroptosis through GSH/GPX4 axis

**Fig. 2:**
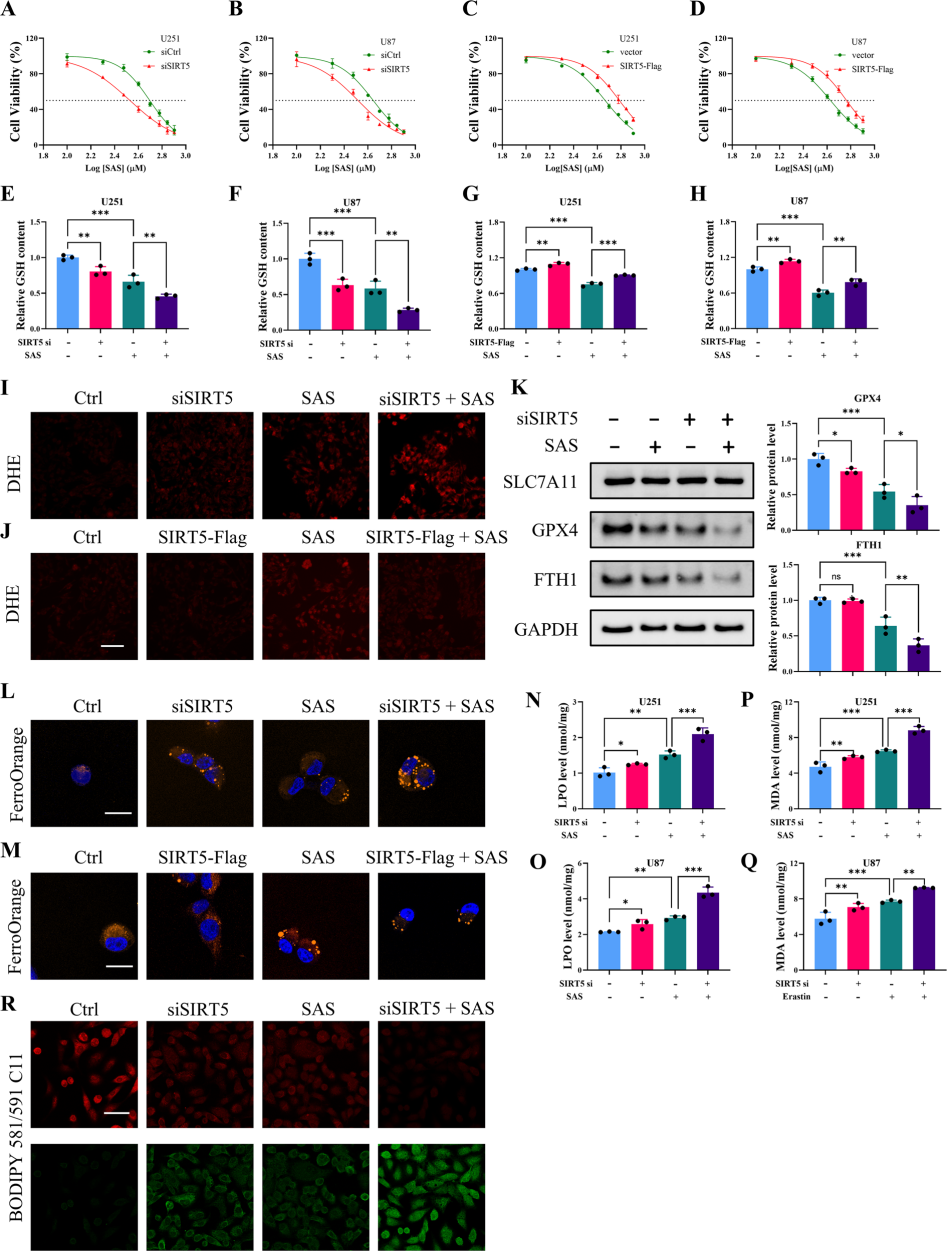
SIRT5 knockdown enhances the sensitivity of glioma cells to ferroptosis through GSH/GPX4 axis. **A**–**D** The IC50 values of SAS in U251 and U87 cells were determined by CCK8 assay following SIRT5 knockdown or SIRT5 overexpression. **E**–**F** Intracellular GSH content was detected in U251 and U87 cells treated with SAS or transfected with SIRT5 siRNA. **G**–**H** Intracellular GSH content was detected in U251 and U87 cells treated with SAS or transfected with SIRT5-Flag plasmid. **I**–**J** ROS levels in U251 cells were detected by DHE staining. Scale bar = 100 μm. **K** Western blot analysis of SLC7A11, GPX4, and FTH1 expression in U251 cells treated with SAS or transfected with SIRT5 siRNA. **L**–**M** Intracellular labile iron levels were detected by FerroOrange staining. Scale bar = 25 µm. **N**–**Q** Intracellular LPO and MDA levels were detected in U251 and U87 cells treated with SAS or transfected with SIRT5 siRNA. **R** Lipid peroxidation was detected by BODIPY 581/591 C11 staining. Scale bar = 50 µm. Data are presented as mean ± SD of three independent experiments. ns *p* > 0.05, *p < 0.01, **p < 0.01, ***p < 0.001.

Glioma cells, due to elevated intracellular Fe²⁺ levels, exhibit susceptibility to ferroptosis [22], making the induction of this form of cell death a potential therapeutic approach for glioma treatment, and SAS has been considered as adjuvant treatment for glioma by activating ferroptosis [23]. Consequently, the involvement of SIRT5, a key component of the cellular antioxidant defense, in glioma’s resistance to ferroptosis was examined. In this study, the IC50 values of SAS were determined to be 496.5 μM and 442.2 μM in U251 and U87 cells, respectively. Following SIRT5 knockdown, these values significantly dropped to 338.5 μM and 329.2 μM (Fig. 2A, B). Treatment with MC3482 also sensitized U251 and U87 cells to SAS (Fig. S2A-B). Conversely, SIRT5 overexpression increased the IC50 of SAS in these cell lines (Fig. 2C, D). To further elucidate the connection between SIRT5 and ferroptosis, critical components of ferroptosis-related signaling pathways were analyzed. It was observed that SIRT5 knockdown led to a significant reduction in intracellular GSH levels in SAS-treated cells, concurrently increasing oxidative stress, whereas SIRT5 overexpression elevated GSH levels and mitigated oxidative stress (Fig. 2E–J). Given that GPX4 plays a pivotal role in neutralizing lipid peroxides during ferroptosis and FTH1 is responsible for iron storage, their expression was detected via western blot analysis. The results showed decreased expression of both GPX4 and FTH1 in SAS-treated cells, which were further downregulated following SIRT5 knockdown (Fig. 2K). Additionally, heightened levels of LPO, MDA, and Fe²⁺, known markers of ferroptosis, were tested. The results revealed that SIRT5 depletion exacerbated SAS-induced Fe²⁺ accumulation, LPO, and MDA production, while SIRT5 overexpression alleviated Fe²⁺ accumulation (Fig. 2L–Q). Furthermore, SIRT5 knockdown intensified SAS-induced lipid peroxidation (Fig. 2R). These findings indicate that SIRT5 facilitates ferroptosis resistance via the GSH/GPX4 axis.

### SIRT5 plays a pivotal role in regulating BCAA metabolism in glioma cells

**Fig. 3:**
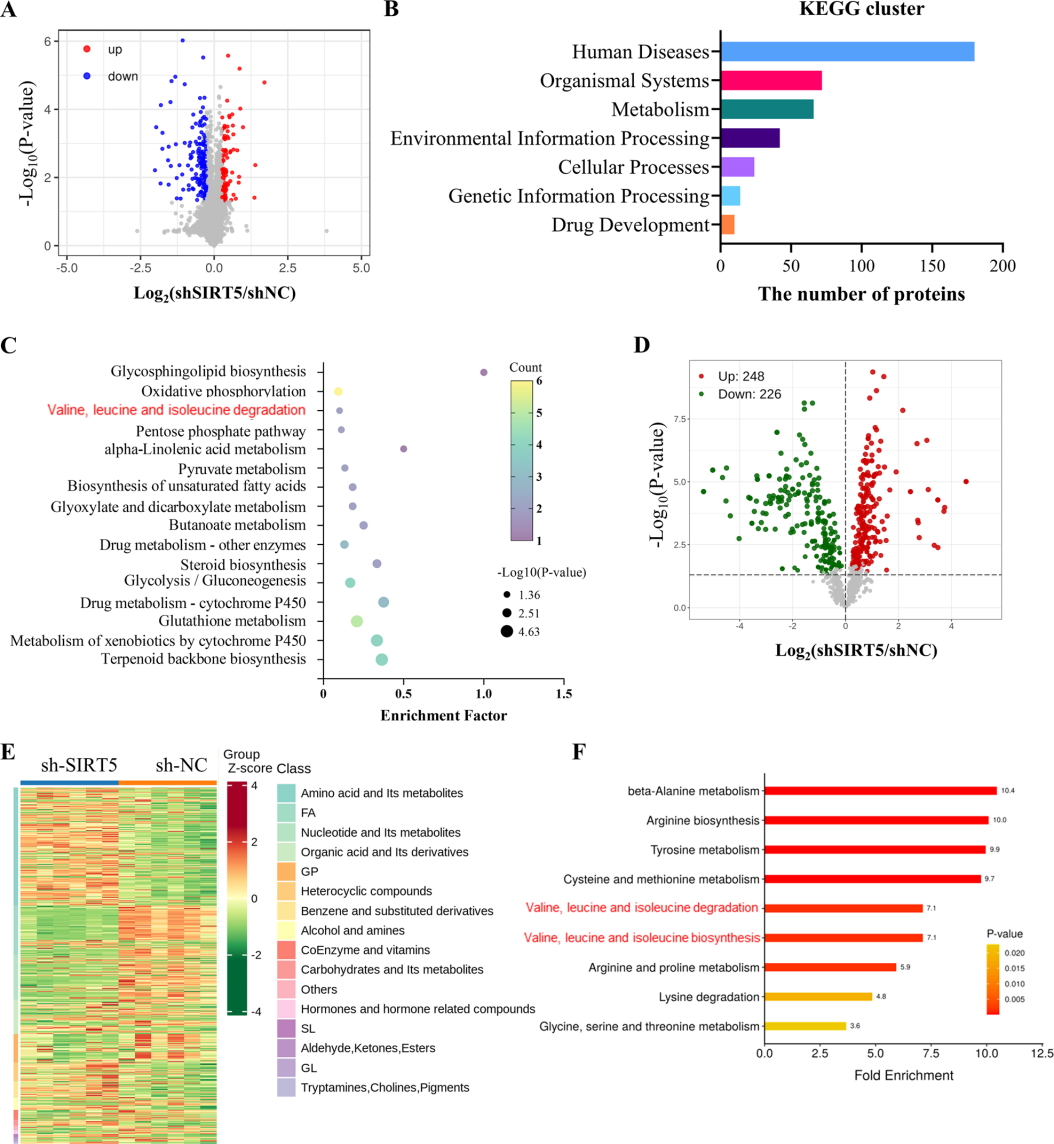
SIRT5 regulates BCAA metabolism in glioma cells. **A** Volcano plot showing proteins differentially expressed between shNC and shSIRT5 U251 cells (*n* = 3). **B**, **C** KEGG pathway analysis of the differentially expressed proteins. **D** Volcano plot depicts differential metabolites between shNC and shSIRT5 U251 cells (*n* = 6). **E** Heatmap visualizes clustering analyses of differential metabolites. **F** KEGG pathway analysis of the differential metabolism.

To further explore the oncogenic mechanism of SIRT5 in glioma, SIRT5 expression was knocked down with lentiviral shRNAs, and a stable SIRT5-knockdown cell line was developed using SIRT5 shRNA-3 (Fig. S3A, B). Subsequent TMT mass spectrometry was conducted to identify proteins differentially expressed between wild-type U251 cells and U251-shSIRT5 cells. Out of the 6633 proteins identified, 112 were upregulated, while 173 exhibited downregulation in SIRT5-knockdown cells (Figs. 3A and S4A). GO enrichment analysis revealed that the differentially expressed proteins were predominantly involved in cellular processes, metabolic processes, and biological regulation (Fig. S4B). Furthermore, KEGG pathway enrichment analysis highlighted significant alterations in glycosphingolipid biosynthesis, oxidative phosphorylation, and valine, leucine, isoleucine degradation upon SIRT5 knockdown (Fig. 3B, C). We further performed metabolomics analysis to elucidate the metabolic alterations associated with SIRT5 knockdown, which identified 474 differentially abundant metabolites (Fig. 3D). Clustering analysis results indicate that most differential metabolites were classified under amino acids and their derivates (Fig. 3E). KEGG analysis further confirmed that ß-alanine metabolism, arginine biosynthesis and tyrosine metabolism were notably affected by SIRT5 knockdown. Importantly, valine, leucine, isoleucine degradation pathways were also enriched (Fig. 3F), which correlates with findings from TMT mass spectrometry. These results indicate that BCAA metabolism is likely a crucial downstream pathway influenced by SIRT5 in glioma cells.

### SIRT5 stabilizes BCAT1 through desuccinylation at K39

**Fig. 4:**
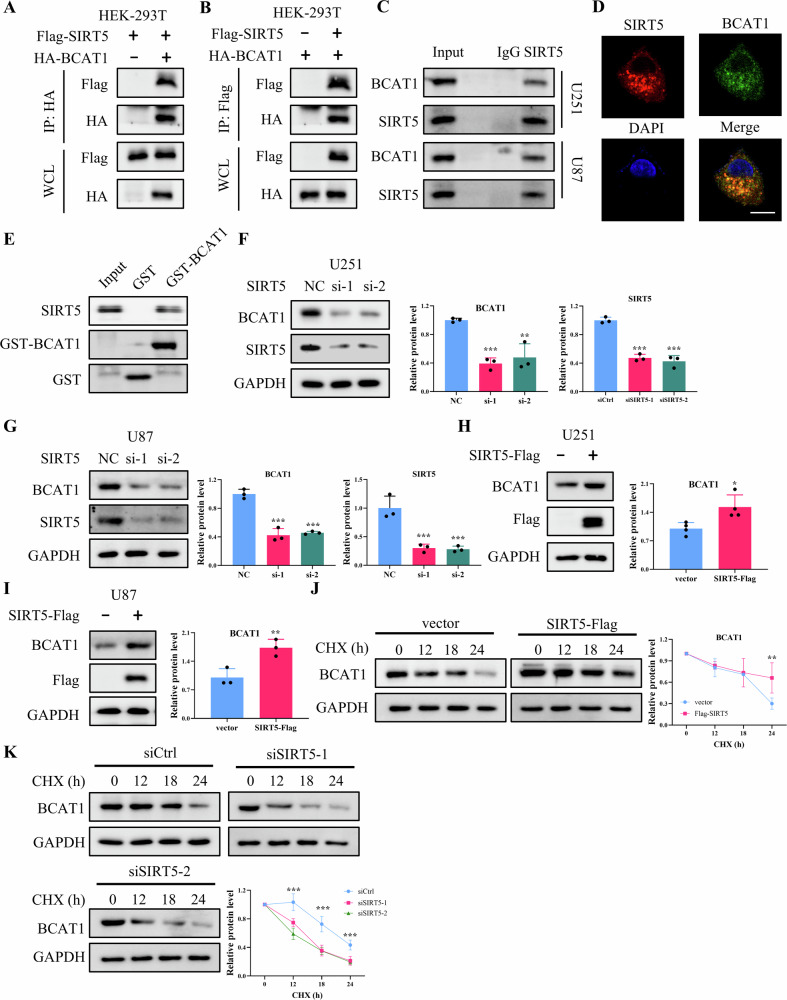
SIRT5 interacts with and stabilizes BCAT1. **A**, **B** The interaction between Flag-tagged SIRT5 and HA-tagged BCAT1 was detected by co-IP and western blot in HEK-293T cells. **C** The interaction between endogenous SIRT5 and BCAT1 in U251 and U87 cells was detected by co-IP and western blot. **D** Immunofluorescence assay of SIRT5 and BCAT1 cellular localization in U251 cells. Scale bar = 10 μm. **E** GST-pull down assay of the interaction between SIRT5 and BCAT1. **F**–**G** Western blot analysis of SIRT5 and BCAT1 expression in U251 and U87 cells transfected with scramble siRNA or SIRT5 siRNA. **H**, **I** Western blot analysis of SIRT5 and BCAT1 expression in U251 and U87 cells transfected with vector or SIRT5-Flag plasmid. **J**, **K** Western blot analysis of BCAT1 expression in U251 cells treated with 50 μg/mL cycloheximide. Data are presented as mean ± SD of three independent experiments. *p < 0.01, **p < 0.01, ***p < 0.001.

BCAT1 catalyzes the initial step of BCAA metabolism and plays a pivotal role in glioma progression [24]. Thus, we sought to determine if SIRT5 modulates BCAA metabolism and exerts its oncogenic functions via BCAT1 regulation. To assess the interaction between SIRT5 and BCAT1, Flag-tagged SIRT5 and HA-tagged BCAT1 were co-expressed in HEK-293T cells. Co-immunoprecipitation experiments demonstrated that SIRT5-Flag and HA-BCAT1interacted with each other (Fig. 4A, B), and similar interaction was observed between endogenous BCAT1 and SIRT5 in U251 and U87 cells (Fig. 4C). Additionally, immunofluorescence assay confirmed that SIRT5 and BCAT1 co-localize in U251 cells (Fig. 4D), and GST-pull down assay further verified their direct interaction in vitro (Fig. 4E).

We then examined whether SIRT5 influences BCAT1 expression at the protein or mRNA level. Western blot analysis revealed that SIRT5 knockdown significantly reduced BCAT1 protein levels in both U251 and U87 cells (Fig. 4F, G), while SIRT5 overexpression elevated BCAT1 protein levels (Fig. 4H, I). Notably, these changes in BCAT1 expression occurred at the protein level, with no significant alterations observed in BCAT1 mRNA levels. (Fig. S5A–D). Furthermore, cycloheximide chase assays demonstrated that SIRT5 overexpression delayed BCAT1 protein turnover, whereas SIRT5 depletion accelerated its degradation (Fig. 4J, K), suggesting that SIRT5 stabilizes BCAT1 in glioma cells.

**Fig. 5:**
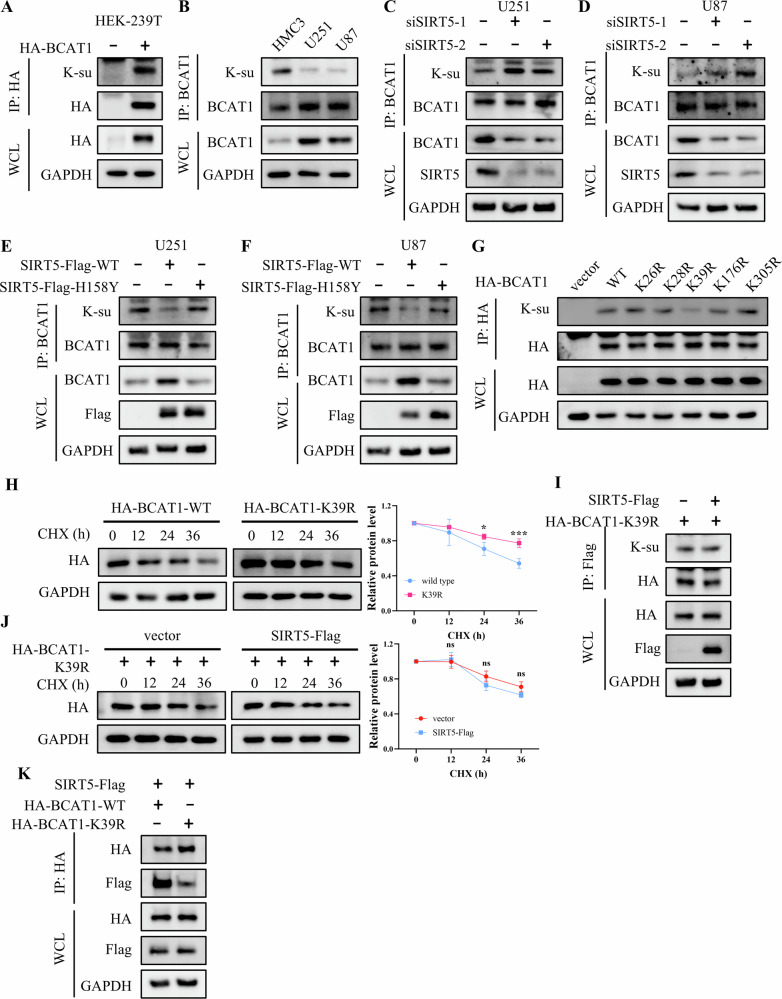
K39 is the primary site in SIRT5-mediated BCAT1 desuccinylation. **A** IP and western blot analysis of BCAT1 succinylation levels in HEK-293T cells transfected with HA-BCAT1 plasmid. **B** IP and western blot analysis of endogenous BCAT1 succinylation levels in HMC3, U87, and U251 cells. **C**, **D** IP and western blot analysis of BCAT1 succinylation levels in U87 and U251 cells transfected with scramble siRNA or SIRT5 siRNA. **E**, **F** IP and western blot analysis of BCAT1 succinylation levels in U87 and U251 cells transfected with vector or SIRT5-Flag plasmid. **G** IP and western blot analysis of the succinylation levels of wild-type BCAT1 and BCAT1 mutant in U251 cells. **H** Western blot analysis of BCAT1 expression in U251 cells treated with 50 μg/mL cycloheximide. **I** IP and western blot analysis of the succinylation levels of BCAT1 K39R mutant in U251 cells. **J** Western blot analysis of BCAT1 expression in U251 cells treated with 50 μg/mL cycloheximide. **K** co-IP and western blot analysis of the interaction between SIRT5-Flag and wild-type BCAT1 or BCAT1 K39R mutant. Data are presented as mean ± SD of three independent experiments. ns *p* > 0.05, *p < 0.01, ***p < 0.001.

Considering SIRT5’s enzymatic role as both deacetylase and dessuccinylase [25], we assessed its effect on BCAT1 acetylation and succinylation. Our results indicated that BCAT1 is subjected to both modifications (Figs. 5A and S6A). However, BCAT1 succinylation was lower in U251 and U87 cells compared to HMC3 cells, while its acetylation levels remained consistent across the cell lines. (Figs. 5B and S6B). Interestingly, SIRT5 knockdown increased BCAT1 succinylation (Fig. 5C, D), while ectopic expression of wild-type SIRT5, but not its enzyme-deficient mutant, lowered BCAT1 succinylation (Fig. 5E, F). Moreover, neither of these interventions affected BCAT1 acetylation (Fig. S6C, D). To pinpoint the key succinylation site, five candidate lysine residues (K26, K28, K39, K176, K305) were predicted using LMSuccSite Platform (Table. S2), and each was mutated to arginine to mimic desuccinylation state. As shown in Fig. 5G, H, the BCAT1 K39R mutant exhibited reduced succinylation and a longer half-life compared to wild-type BCAT1, suggesting that K39 is the primary succinylation site on BCAT1. We then explored whether SIRT5 regulated BCAT1 expression through desuccinylation at K39. The results revealed that SIRT5 overexpression had no impact on the succinylation level or half-life of BCAT1 K39R mutant (Fig. 5I, J). And SIRT5 showed a weaker interaction with the K39R mutant than with wild-type BCAT1 (Fig. 5K), indicating that SIRT5 stabilizes BCAT1 via K39 desuccinylation.

### SIRT5-mediated BCAT1 desuccinylation suppresses its ubiquitin-proteasome degradation

**Fig. 6:**
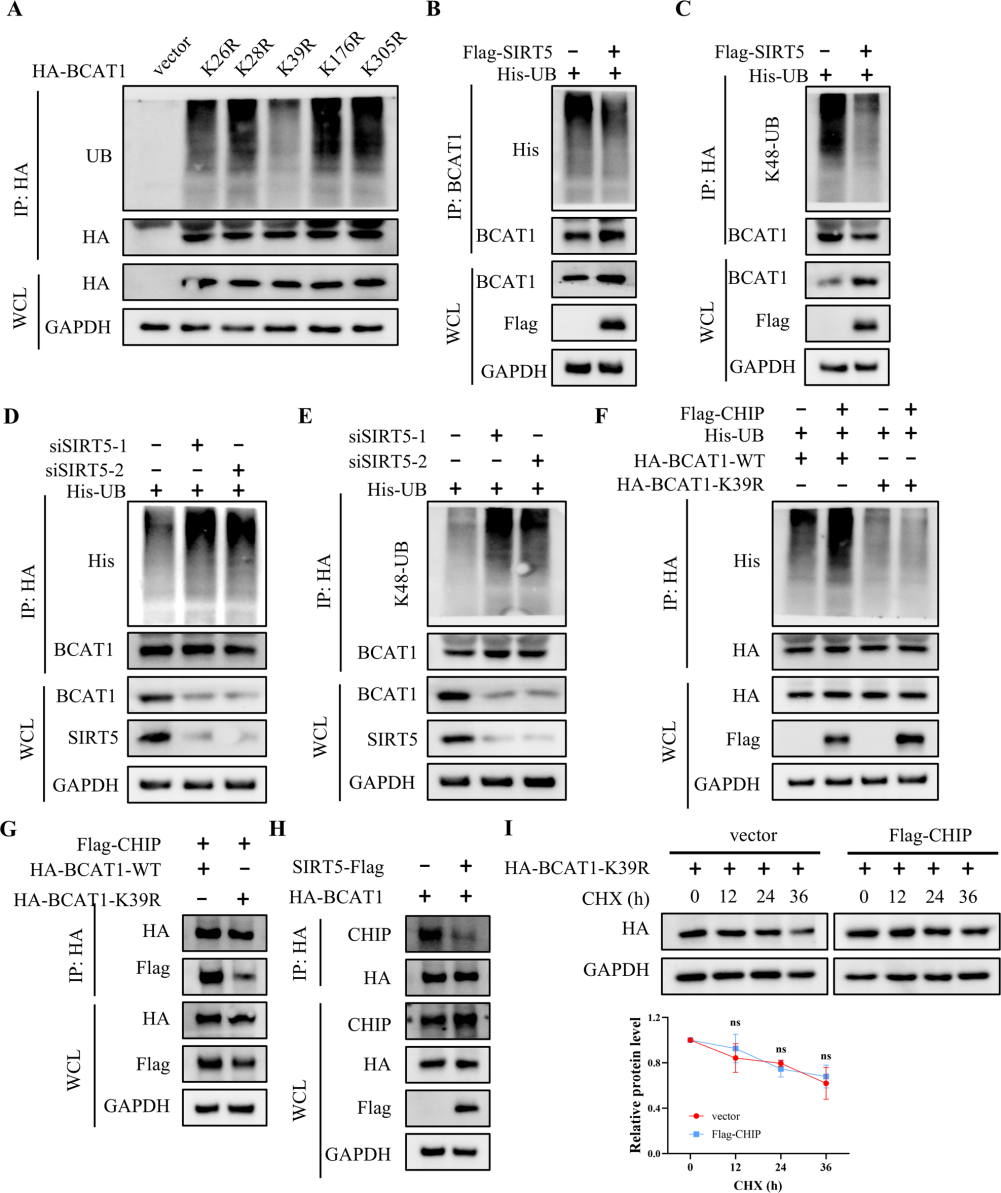
SIRT5-mediated BCAT1 desuccinylation suppresses its ubiquitin-proteasome degradation. **A** IP and western blot analysis of the ubiquitination levels of wild-type BCAT1 and BCAT1 mutant. **B**, **C** IP, and western blot analysis of wild-type and K48-linkage BCAT1 ubiquitination levels in U251 cells transfected with vector or SIRT5-Flag plasmid. **D**, **E** IP and western blot analysis of wild-type and K48-linkage BCAT1 ubiquitination levels in U251 cells transfected with scramble siRNA or SIRT5 siRNA. **F** IP and western blot analysis of the ubiquitination levels of wild-type BCAT1 and BCAT1 K39R mutant in U251 cells transfected with vector or Flag-CHIP plasmid. **G**, **H** co-IP and western blot analysis of the interaction between CHIP and BCAT1 in U251 cells. **I** Western blot analysis of BCAT1 expression in U251 cells treated with 50 μg/mL cycloheximide. Data are presented as mean ± SD of three independent experiments. ns *p* > 0.05.

Our previous study has shown that E3 ligase CHIP mediates BCAT1 ubiquitination, promoting its degradation [26]. We sought to investigate whether the stabilization of BCAT1 by SIRT5 is associated with its ubiquitination status. Western blot analysis revealed that the BCAT1 K39R mutant exhibited lower levels of ubiquitination compared to wild-type BCAT1 (Fig. 6A). Additionally, SIRT5 overexpression was found to decrease both wild-type and K48-linkage BCAT1 ubiquitination (Fig. 6B, C). In contrast, depletion of SIRT5 increased these forms of BCAT1 ubiquitination (Fig. 6D, E). To further explore the interplay between BCAT1 succinylation and ubiquitination, Flag-tagged CHIP was co-transfected with wild-type BCAT1 or BCAT1 K39R mutant. Western blot analysis indicated that CHIP specifically increased the ubiquitination of wild-type BCAT1, while having no significant effect on K39R mutant (Fig. 6F). Moreover, CHIP interacted less with the BCAT1 K39R mutant compared to wild-type BCAT1, and SIRT5 overexpression reduced the interaction between CHIP and BCAT1 (Fig. 6G, H). Notably, CHIP overexpression did not significantly affect the half-life of the BCAT1 K39R mutant (Fig. 6I). In summary, these findings suggest that SIRT5-mediated desuccinylation of BCAT1 at K39 inhibits its interaction with CHIP, thereby preventing ubiquitin-proteasome degradation of BCAT1.

### SIRT5 stabilizes BCAT1 to promote cell proliferation and tumor growth

**Fig. 7:**
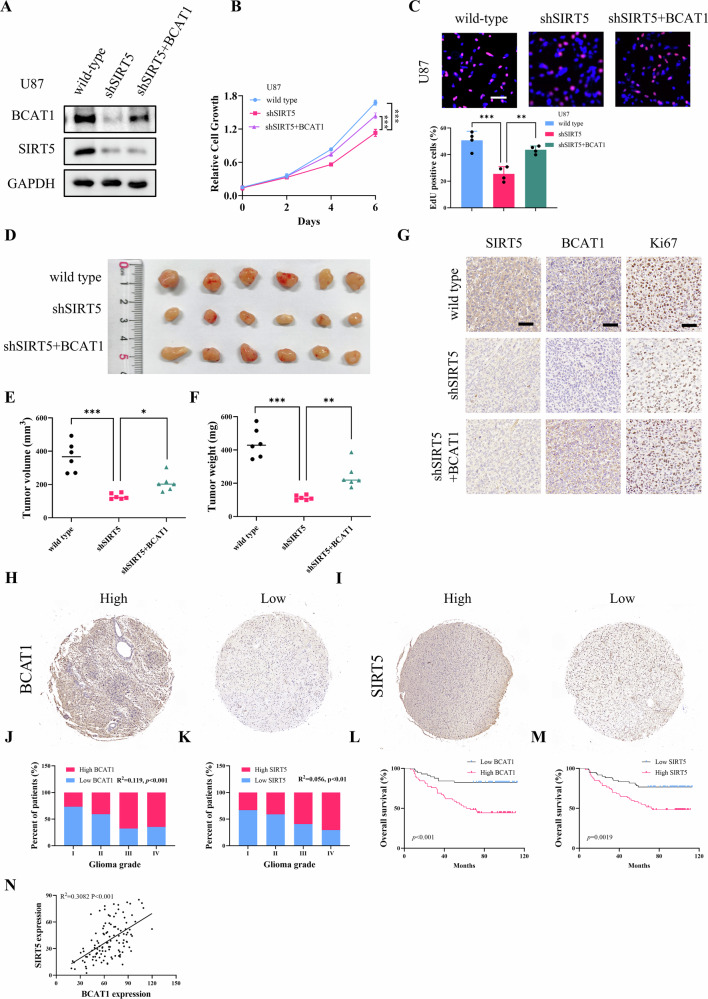
SIRT5 stabilizes BCAT1 to promotes cell proliferation and tumor growth in vitro and in vivo. **A** Western blot analysis of BCAT1 and SIRT5-Flag expression in wild-type U87 cells, U87-shSIRT5 cells, and U87-shSIRT5 + BCAT1 cells. **B**, **C** Crystal violet staining and EdU staining were performed to evaluate the proliferative ability of wild-type U87 cells, U87-shSIRT5 cells, and U87-shSIRT5 + BCAT1 cells. Scale bar = 100 μm. **D**–**F** Photographs, volume, and weight of the xenografted tumors. **G** IHC staining of the xenografted tumor sections in each group. Scale bar = 50 μm. Data are presented as mean ± SD (*n* = 6). **p < 0.01, ***p < 0.001. **H**, **I** Representative IHC images of glioma microarray showing SIRT5 and BCAT1 expression. **J**–**K** Correlation analysis of SIRT5 and BCAT1 expression with glioma grades. **L**, **M**) Kaplan–Meier survival curve of glioma patients based on SIRT5 and BCAT1 expression. **N** Correlation between SIRT5 and BCAT1 expression in glioma tissue microarrays.

To further validate the functional role of SIRT5/BCAT1 axis in glioma, stable cell lines were generated in which SIRT5 was knocked down, and BCAT1 was overexpressed in U87 glioma cells (Fig. 7A). Cell proliferation assay and EdU staining revealed that compared to wild-type U87 cells, those with SIRT5 knockdown (U87-shSIRT5) exhibited a significantly reduced proliferation rate, which was restored upon BCAT1 overexpression (U87-shSIRT5 + BCAT1) (Fig. 7B, C). Similarly, in a xenograft mouse model, mice injected with U87-shSIRT5 cells showed notably smaller tumor volumes and reduced tumor weights compared to those xenografted with wild-type U87 cells. However, tumor growth was restored in mice xenografted with U87-shSIRT5 + BCAT1 cells (Fig. 7D–F). IHC staining for Ki67, a marker of cell proliferation, confirmed lower expression in the shSIRT5 group but elevated levels in the shSIRT5 + BCAT1 group (Fig. 7G). Together, these results clearly indicate that SIRT5 promotes glioma progression by regulating BCAT1.

### High SIRT5 and BCAT1 expression correlates with poor prognosis of glioma patients

To better understand the clinical relevance of SIRT5 and BCAT1 in glioma, tissue microarrays were analyzed for their expression levels in glioma patient samples (Fig. 7H, I). The results demonstrated that both SIRT5 and BCAT1 were significantly upregulated in patients with advanced tumor grades (Fig. 7J, K). Survival analysis indicated that patients with higher levels of either SIRT5 or BCAT1 expression had notably shorter overall survival (Fig. 7L, M). Furthermore, a strong positive correlation was identified between SIRT5 and BCAT1 expression within the tissue microarray samples (Fig. 7N). These findings suggest that the elevated expression of SIRT5 and BCAT1 contributes to glioma progression and may serve as valuable prognostic markers for glioma patients.

### SIRT5-mediated BCAT1 stabilization facilitates ferroptosis resistance

**Fig. 8:**
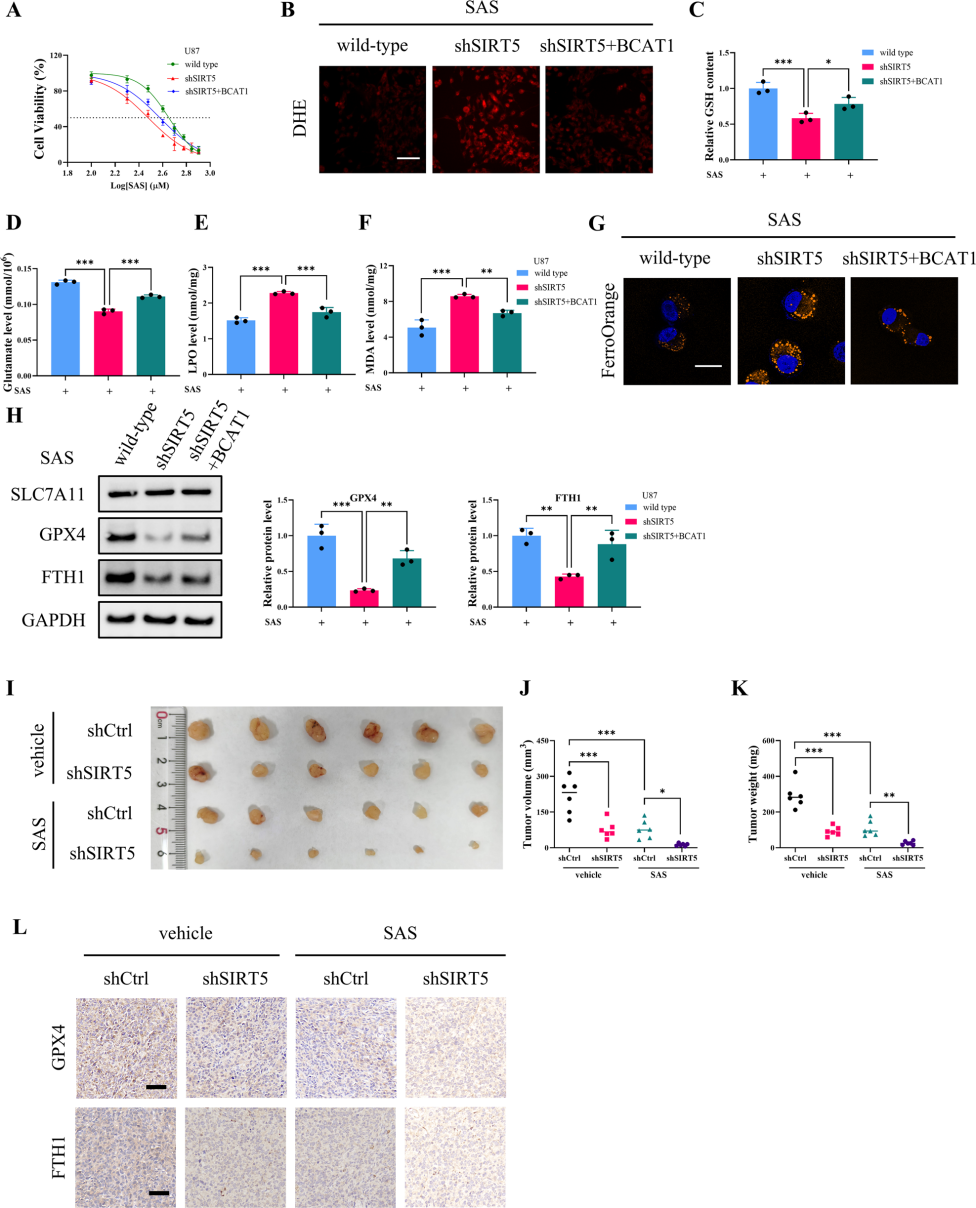
SIRT5-mediated BCAT1 stabilization facilitates ferroptosis resistance. **A** CCK8 assay was performed to evaluate the IC50 values for SAS in wild-type U87 cells, U87-shSIRT5 cells, and U87-shSIRT5 + BCAT1 cells. **B** DHE staining was performed to assess ROS levels in wild-type U87 cells, U87-shSIRT5 cells, and U87-shSIRT5 + BCAT1 cells treated with SAS. Scale bar = 100 μm. **C**–**F** Intracellular GSH, glutamate, LPO, and MDA levels were detected in wild-type U87 cells, U87-shSIRT5 cells, and U87-shSIRT5 + BCAT1 cells treated with SAS. **G** Intracellular labile iron levels were detected by FerroOrange staining. Scale bar = 25 µm. **H** Western blot analysis of SLC7A11, GPX4, and FTH1 expression wild-type U87 cells, U87-shSIRT5 cells, and U87-shSIRT5 + BCAT1 cells treated with SAS. **I**–**K** Photographs, volume, and weight of the xenografted tumors. **L** IHC staining of the xenografted tumor sections in each group. Scale bar = 50 μm. Data are presented as mean ± SD (*n* = 6). *p < 0.01, **p < 0.01, ***p < 0.001.

We further examined the impact of SIRT5/BCAT1 axis on ferroptosis resistance in glioma cells. U87-shSIRT5 cells exhibited enhanced sensitivity to SAS, and ectopic expression of BCAT1 restored resistance to SAS in SIRT5-knockdown U87 cells (Fig. 8A). Upon SAS treatment, U87-shSIRT5 cells exhibited increased ROS levels and reduced GSH and glutamate levels in comparison to wild-type U87 and U87-shSIRT5 + BCAT1 cells (Fig. 8B–D). In addition, the levels of LPO, MDA, and Fe²⁺, all markers of ferroptosis, were significantly elevated in U87-shSIRT5 cells, which were ameliorated by ectopic expression of BCAT1 (Fig. 8E–G). Moreover, the expression of GPX4 and FTH1 was markedly decreased in U87-shSIRT5 cells (Fig. 8H). In in vivo experiments, mice xenografted with wild-type U87 cells or U87-shSIRT5 cells were intraperitoneally injected with SAS every other day. Results demonstrated that SIRT5 depletion potentiated SAS’s anti-tumor efficacy (Fig. 8I–K). IHC staining also revealed decreased expression of GPX4 and FTH1in the tumors from U87-shSIRT5 cells administrated with SAS (Fig. 8L). In sum, these data suggest that SIRT5/BCAT1 axis effectively enhances ferroptosis resistance in glioma.

## Discussion

SIRT5 is emerging as a promising target for cancer therapy due to its critical role in various metabolic pathways [25]. Interestingly, SIRT5 demonstrates a dual function in the context of cancers, acting as a tumor suppressor in renal cell carcinoma and pancreatic cancer, while exhibiting oncogenic properties in breast cancer and non-small cell lung cancer [27]. The expression and performance of SIRT5 in glioma, however, remain ambiguous and disputed. While certain studies report that SIRT5 expression is elevated in glioma tissue compared to adjacent non-cancerous tissue [28], Tang et al., in contrast, associate higher SIRT5 expression with improved survival rates in glioma patients [29]. In our research, we observe increased SIRT5 expression in U251 and U87 cells compared to HMC3 cells, and in glioma tissues compared to normal brain tissues. Through microarray analysis, we establish that SIRT5 expression positively correlates with glioma grade and unfavorable prognosis. Functional experiments reveal that overexpressing SIRT5 enhances glioma cell proliferation, whereas SIRT5 knockdown impairs cell proliferation and tumor growth both in vitro and in vivo. Overall, our findings indicate that SIRT5 functions as an oncogene in glioma.

Although certain ferroptosis inducers show therapeutic promise in various cancer types, sulfasalazine remains the only reagent that has been under evaluation for glioma treatment in clinical trials [30, 31]. However, several unsolved obstacles remain that limit its clinical use. For instance, glioma cells have a higher level of GSH than normal brain cells [32], making it insensitive to ferroptosis inducers. Indeed, in vitro studies have shown that SAS only becomes gliomatoxic at concentrations exceeding 200 μM [21]. Therefore, overcoming ferroptosis insensitivity remains a key research focus. SIRT5 has been shown to enhance cellular antioxidant defenses by promoting IDH2 desuccinylation and G6PD deglutarylation, as well as by desuccinylating SOD1, thus mitigating oxidative stress [33, 34]. These mechanisms indicate that SIRT5 may prevent ferroptosis. Recent findings suggest that SIRT5 regulates ferroptosis through the Nrf2/HO-1 pathway in ischemic stroke models [35]. SIRT5 also regulates glutamine metabolism [13], which is a key source of glutamate and GSH. However, no evidence has linked SIRT5 to ferroptosis insensitivity in cancer. Our study demonstrates that high SIRT5 expression correlates ferroptosis insensitivity in glioma cells. Specifically, SIRT5 knockdown decreases the IC50 of SAS to in glioma cells. Mechanistically, GPX4 and FTH1 expression are markedly downregulated following SAS treatment in SIRT5-knockdown cells, resulting in Fe^2+^ accumulation, ROS production, and lipid peroxidation. These findings provide strong evidence that SIRT5 acts as a ferroptosis repressor in glioma.

Enhanced BCAA metabolism is a hallmark of glioma, and its inhibition triggers apoptosis in glioma cells both in vitro and in vivo [36]. Our proteomic and metabolomic analysis indicates a relationship between SIRT5 and BCAA metabolism. BCAT1, which catalyzes the initial step in BCAA metabolism, is overexpressed in most cancer types, including glioma, where its high expression correlates with unfavorable patient prognosis [37]. It has been reported that increased BCAT1 expression boosts glutamate levels, enhancing GSH production and supporting GPX4 activity, which protects against lipid peroxidation and ferroptosis [38]. Post-translational modification plays crucial role in the regulation of BCAT1. Phosphorylation and palmitoylation regulate BCAT1’s activity and intracellular localization [39]. Additionally, CHIP-mediated ubiquitination of BCAT1 leads to its proteasomal degradation, reducing cell proliferation and resistance to temozolomide in glioma [26]. In this study, we reveal for the first time that BCAT1 undergoes both acetylation and succinylation in glioma cells. Glioma cells exhibit reduced succinylation of BCAT1, which stabilizes the protein. We further identify BCAT1 as a novel desuccinylation substrate of SIRT5, with SIRT5 directly binding to BCAT1 and desuccinylating it at K39. This desuccinylation suppresses the interaction between BCAT1 and the E3 ligase CHIP, preventing BCAT1 degradation. These findings reveal a novel post-modification cross-talk on BCAT1. Furthermore, BCAT1 overexpression rescues the proliferation inhibition observed in SIRT5 knockdown cells. Following SAS treatment, BCAT1 overexpression restores intracellular GSH levels and mitigates oxidative stress, thereby conferring resistance to SAS-induced ferroptosis. To further study the role of SIRT5 and BCAT1 in clinical, our data shows a correlation between SIRT5 and BCAT1 expression in glioma microarray, with higher expression levels predicting higher glioma grade and worse clinical outcome.

In conclusion, our study proposes a hierarchical model in which elevated SIRT5 expression stabilizes BCAT1 through desuccinylation, promoting BCAA metabolism and glioma cell proliferation. Additionally, the SIRT5/BCAT1 axis increases intracellular GSH levels, alleviates ROS production, and inhibits lipid peroxidation, contributing to ferroptosis insensitivity in glioma cells. Thus, our study is the first to highlight the crucial role of SIRT5 in the ferroptosis resistance, emphasizing its therapeutic potential for glioma patients.

## Supplementary information

Supplemental Figures

Table S1

Table S2

Raw WB Data

## Data Availability

The data that support the findings of this study are available from the corresponding author upon reasonable request.

## References

[1] Bray F, Laversanne M, Sung H, Ferlay J, Siegel RL, Soerjomataram I, et al. Global cancer statistics 2022: GLOBOCAN estimates of incidence and mortality worldwide for 36 cancers in 185 countries. CA A Cancer J Clin. 2024;74:229–63. 10.3322/caac.21834 38572751

[2] Liang T, Gu L, Kang X, Li J, Song Y, Wang Y, et al. Programmed cell death disrupts inflammatory tumor microenvironment (TME) and promotes glioblastoma evolution. Cell Commun Signal. 2024;22:333. 38890642 10.1186/s12964-024-01602-0 PMC11184850

[3] Bi J, Chowdhry S, Wu S, Zhang W, Masui K, Mischel PS. Altered cellular metabolism in gliomas — an emerging landscape of actionable co-dependency targets. Nature Rev Cancer. 2019;20:57–70. 31806884 10.1038/s41568-019-0226-5

[4] Chang H-C, Guarente L. SIRT1 and other sirtuins in metabolism. Trends Endocrinol Metab. 2014;25:138–45. 24388149 10.1016/j.tem.2013.12.001 PMC3943707

[5] Rardin Matthew J, He W, Nishida Y, Newman John C, Carrico C, Danielson Steven R, et al. SIRT5 regulates the mitochondrial lysine succinylome and metabolic networks. Cell Metab. 2013;18:920–33. 24315375 10.1016/j.cmet.2013.11.013 PMC4105152

[6] Wang G, Meyer JG, Cai W, Softic S, Li ME, Verdin E, et al. Regulation of UCP1 and mitochondrial metabolism in brown adipose tissue by reversible succinylation. Mol Cell. 2019;74:844–57.e847. 31000437 10.1016/j.molcel.2019.03.021 PMC6525068

[7] Chen XF, Tian MX, Sun RQ, Zhang ML, Zhou LS, Jin L, et al. SIRT5 inhibits peroxisomal ACOX1 to prevent oxidative damage and is downregulated in liver cancer. EMBO Rep. 2018:19:e45124. 10.15252/embr.201745124 PMC5934778 29491006

[8] Du J, Zhou Y, Su X, Yu JJ, Khan S, Jiang H, et al. Sirt5 Is a NAD-dependent protein lysine demalonylase and desuccinylase. Science. 2011;334:806–9. 22076378 10.1126/science.1207861 PMC3217313

[9] Wang Y, Chen H, Zha X. Overview of SIRT5 as a potential therapeutic target: Structure, function and inhibitors. Eur J Med Chem. 2022;236:114363. 35436671 10.1016/j.ejmech.2022.114363

[10] Wang C, Zhang C, Li X, Shen J, Xu Y, Shi H, et al. CPT1A-mediated succinylation of S100A10 increases human gastric cancer invasion. J Cell Mol Med. 2018;23:293–305. 30394687 10.1111/jcmm.13920 PMC6307794

[11] Hu T, Shukla SK, Vernucci E, He C, Wang D, King RJ, et al. Metabolic rewiring by loss of Sirt5 promotes Kras-induced pancreatic cancer progression. Gastroenterology. 2021;161:1584–1600. 34245764 10.1053/j.gastro.2021.06.045 PMC8546779

[12] Yang X, Wang Z, Li X, Liu B, Liu M, Liu L, et al. SHMT2 desuccinylation by SIRT5 drives cancer cell proliferation. Cancer Res. 2018;78:372–86. 29180469 10.1158/0008-5472.CAN-17-1912

[13] Polletta L, Vernucci E, Carnevale I, Arcangeli T, Rotili D, Palmerio S, et al. SIRT5 regulation of ammonia-induced autophagy and mitophagy. Autophagy. 2015;11:253–70. 25700560 10.1080/15548627.2015.1009778 PMC4502726

[14] Dixon Scott J, Lemberg Kathryn M, Lamprecht Michael R, Skouta R, Zaitsev Eleina M, Gleason Caroline E, et al. Ferroptosis: an iron-dependent form of nonapoptotic cell death. Cell. 2012;149:1060–72. 22632970 10.1016/j.cell.2012.03.042 PMC3367386

[15] Jiang X, Stockwell BR, Conrad M. Ferroptosis: mechanisms, biology and role in disease. Nat Rev Mol Cell Biol. 2021;22:266–82. 33495651 10.1038/s41580-020-00324-8 PMC8142022

[16] Walker MC, van der Donk WA. The many roles of glutamate in metabolism. J Ind Microbiol Biotechnol. 2016;43:419–30. 26323613 10.1007/s10295-015-1665-y PMC4753154

[17] Chen X, Kang R, Kroemer G, Tang D. Broadening horizons: the role of ferroptosis in cancer. Nat Rev Clin Oncol. 2021;18:280–96. 33514910 10.1038/s41571-020-00462-0

[18] Cheng J, Fan YQ, Liu BH, Zhou H, Wang JM, Chen QX. ACSL4 suppresses glioma cells proliferation via activating ferroptosis. Oncol Rep. 2020;43:147–58. 31789401 10.3892/or.2019.7419 PMC6912066

[19] Wang Z, Ding Y, Wang X, Lu S, Wang C, He C, et al. Pseudolaric acid B triggers ferroptosis in glioma cells via activation of Nox4 and inhibition of xCT. Cancer Lett. 2018;428:21–33. 29702192 10.1016/j.canlet.2018.04.021

[20] Chung WJ, Lyons SA, Nelson GM, Hamza H, Gladson CL, Gillespie GY, et al. Inhibition of cystine uptake disrupts the growth of primary brain tumors. J Neurosci. 2005;25:7101–10. 16079392 10.1523/JNEUROSCI.5258-04.2005 PMC2681064

[21] Sehm T, Fan Z, Ghoochani A, Rauh M, Engelhorn T, Minakaki G, et al. Sulfasalazine impacts on ferroptotic cell death and alleviates the tumor microenvironment and glioma-induced brain edema. Oncotarget. 2016;7:36021–33. 27074570 10.18632/oncotarget.8651 PMC5094980

[22] Chirasani SR, Markovic DS, Synowitz M, Eichler SA, Wisniewski P, Kaminska B, et al. Transferrin-receptor-mediated iron accumulation controls proliferation and glutamate release in glioma cells. J Mol Med. 2008;87:153–67. 19066835 10.1007/s00109-008-0414-3

[23] Sontheimer H, Bridges RJ. Sulfasalazine for brain cancer fits. Expert Opin Investig Drugs. 2012;21:575–8. 22404218 10.1517/13543784.2012.670634 PMC3644176

[24] Tönjes M, Barbus S, Park YJ, Wang W, Schlotter M, Lindroth AM, et al. BCAT1 promotes cell proliferation through amino acid catabolism in gliomas carrying wild-type IDH1. Nat Med. 2013;19:901–8. 23793099 10.1038/nm.3217 PMC4916649

[25] Kumar S, Lombard DB. Functions of the sirtuin deacylase SIRT5 in normal physiology and pathobiology. Crit Rev Biochem Mol Biol. 2018;53:311–34. 29637793 10.1080/10409238.2018.1458071 PMC6233320

[26] Lu Z, Wang XY, He KY, Han XH, Wang X, Zhang Z, et al. CHIP-mediated ubiquitin degradation of BCAT1 regulates glioma cell proliferation and temozolomide sensitivity. Cell Death Dis. 2024;15:538. 39075053 10.1038/s41419-024-06938-6 PMC11286746

[27] Yihan L, Xiaojing W, Ao L, Chuanjie Z, Haofei W, Yan S, et al. SIRT5 functions as a tumor suppressor in renal cell carcinoma by reversing the Warburg effect. J Transl Med. 2021;19:521. 34930316 10.1186/s12967-021-03178-6 PMC8690424

[28] Xuan F, Zhang Z, Liu K, Gong H, Liang S, Zhao Y, et al. Constructing a signature based on the SIRT family to help the prognosis and treatment sensitivity in glioma patients. Front Genet. 2022;13:1035368. 36568393 10.3389/fgene.2022.1035368 PMC9780371

[29] Tang W, Chen B, Leung GK-K, Kiang KM. Sirtuin 5 (SIRT5) suppresses tumor growth by regulating mitochondrial metabolism and synaptic remodeling in gliomas. Int J Mol Sci. 2024;25:9125. 10.3390/ijms25169125 PMC11354685 39201811

[30] Skeie BS, Bragstad S, Sarowar S, Behbahani M, Filippi C, Knisely J, et al. Ctni-40. phase I trial of sulfasalazine combined with stereotactic radiosurgery for recurrent glioblastoma: study protocol for Nct04205357. Neuro Oncol. 2022;24:vii80–1.

[31] Robe PA, Martin D, Albert A, Deprez M, Chariot A, Bours V. A phase 1–2, prospective, double blind, randomized study of the safety and efficacy of Sulfasalazine for the treatment of progressing malignant gliomas: study protocol of [ISRCTN45828668]. BMC Cancer. 2006;6:29. 16448552 10.1186/1471-2407-6-29 PMC1368982

[32] Ogunrinu TA, Sontheimer H. Hypoxia increases the dependence of glioma cells on glutathione. J Biol Chem. 2010;285:37716–24. 20858898 10.1074/jbc.M110.161190 PMC2988376

[33] Zhou L, Wang F, Sun R, Chen X, Zhang M, Xu Q, et al. SIRT5 promotes IDH2 desuccinylation and G6PD deglutarylation to enhance cellular antioxidant defense. EMBO Rep. 2016;17:811–22. 27113762 10.15252/embr.201541643 PMC5278614

[34] Lin Z-F, Xu H-B, Wang J-Y, Lin Q, Ruan Z, Liu F-B, et al. SIRT5 desuccinylates and activates SOD1 to eliminate ROS. Biochem Biophys Res Commun. 2013;441:191–5. 24140062 10.1016/j.bbrc.2013.10.033

[35] Li J, Wei G, Song Z, Chen Z, Gu J, Zhang L, et al. SIRT5 regulates ferroptosis through the Nrf2/HO-1 signaling axis to participate in ischemia-reperfusion injury in ischemic stroke. Neurochem Res. 2024;49:998–1007. 38170384 10.1007/s11064-023-04095-4

[36] Lu Z, Sun GF, He KY, Zhang Z, Han XH, Qu XH, et al. Targeted inhibition of branched-chain amino acid metabolism drives apoptosis of glioblastoma by facilitating ubiquitin degradation of Mfn2 and oxidative stress. Biochim Biophys Acta Mol Basis Dis. 2024;1870:167220. 38718847 10.1016/j.bbadis.2024.167220

[37] Li GS, Huang HQ, Liang Y, Pang QY, Sun HJ, Huang ZG, et al. BCAT1: a risk factor in multiple cancers based on a pan-cancer analysis. Cancer Med. 2022;11:1396–412. 34984849 10.1002/cam4.4525 PMC8894718

[38] Liu N, Li C, Yan C, Yan HC, Jin BX, Yang HR, et al. BCAT1 alleviates early brain injury by inhibiting ferroptosis through PI3K/AKT/mTOR/GPX4 pathway after subarachnoid hemorrhage. Free Radic Biol Med. 2024;222:173–86. 38871197 10.1016/j.freeradbiomed.2024.05.045

[39] Harris M, El Hindy M, Usmari-Moraes M, Hudd F, Shafei M, Dong M, et al. BCAT-induced autophagy regulates Abeta load through an interdependence of redox state and PKC phosphorylation-implications in Alzheimer’s disease. Free Radic Biol Med. 2020;152:755–66. 31982508 10.1016/j.freeradbiomed.2020.01.019

