# Supplementary material for: SIRT5-mediated BCAT1 desuccinylation and stabilization leads to ferroptosis insensitivity and promotes cell proliferation in glioma
Source: Cell Death Dis. 2025 Apr 7;16(1):261. doi: 10.1038/s41419-025-07626-9 (PMC11977203; doi:10.1038/s41419-025-07626-9)
Supplement: Supplementary file 1 — Supplemental Figures [file 41419_2025_7626_MOESM1_ESM.pdf]

Supplemental Figure 1

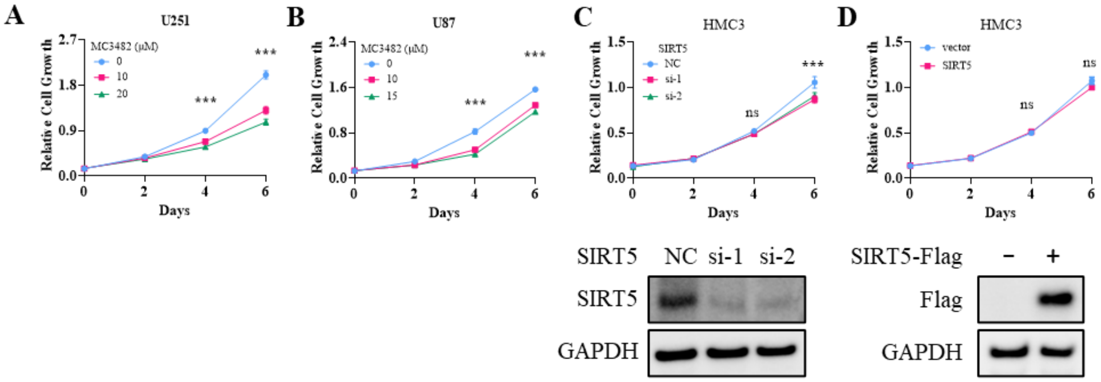

Supplemental Figure 1. Role of SIRT5 in the proliferation of glioma cells and HMC3 cells. (A-B) UU251 and U87 cells were treated with various concentrations of MC3482, and cell proliferation was detected by crystal violet staining. (C-D) Crystal violet staining was performed to evaluate the effect of SIRT5 knockdown or overexpression on the proliferative ability of HMC3 cells. Data are presented as mean ± SD (n=3). ns $p>0.05$, ***$p<0.001$.

Supplemental Figure 2

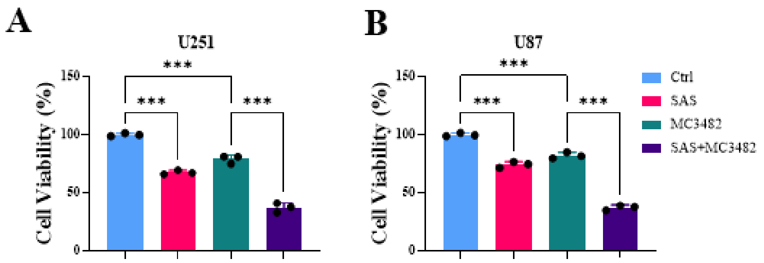

Supplemental Figure 2. MC3482 sensitizes glioma cells to SAS. (A-B) U251 and U87 cells were treated with SAS in the presence or absence of MC3482, and CCK8 assay was performed. Data are presented as mean ± SD (n=3). ***p<0.001.

Supplemental Figure 3

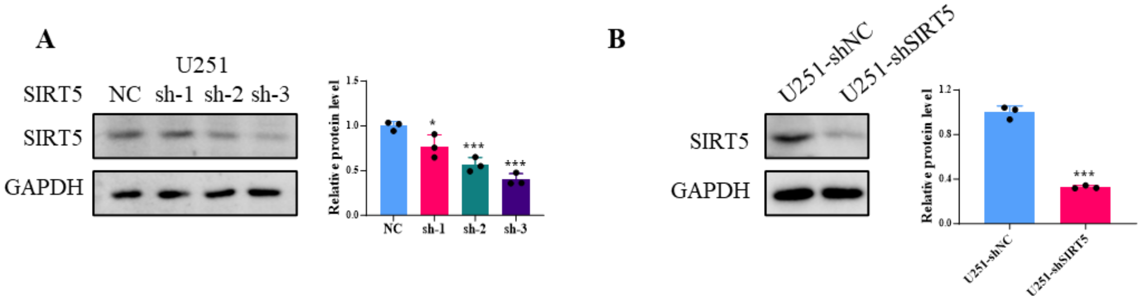

Supplemental Figure 3. Generation of SIRT5 knockdown U251 stable cell line. (A) Western blot analysis of SIRT5 expression in U251 cells transduced with SIRT5 shRNA lentiviral stocks. (B) Western blot analysis of SIRT5 expression in wild-type U251 cells and U251-shSIRT5 cells. Data are presented as mean ± SD (n=3). **p*<0.01, ****p*<0.001.

Supplemental Figure 4

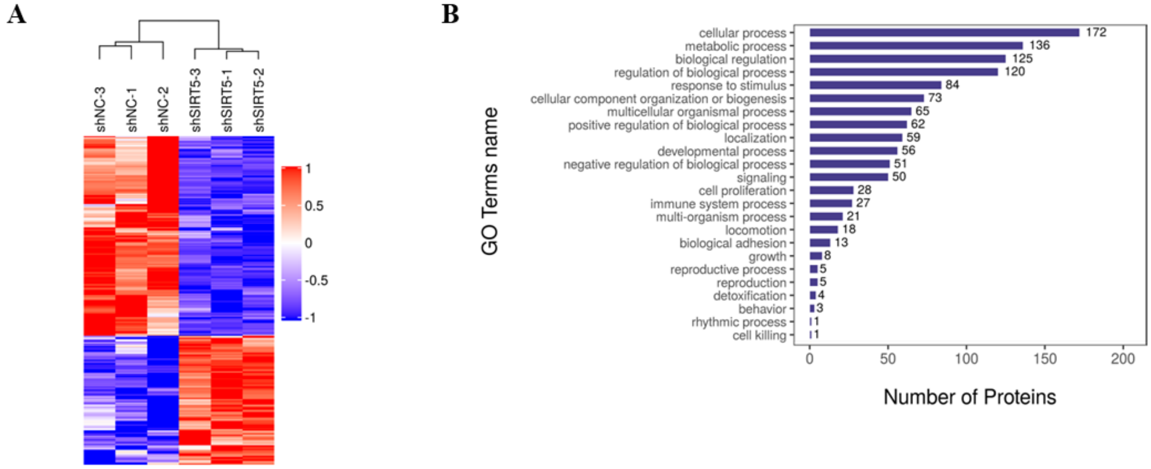

Supplemental Figure 4. Proteomics analysis comparing wild-type U251 cells to U251-shSIRT5 cells. (A) Heatmap showing a visualization of clustering analyses of differentially expressed proteins. (B) KEGG pathway analysis of the differentially expressed proteins.

Supplemental Figure 5

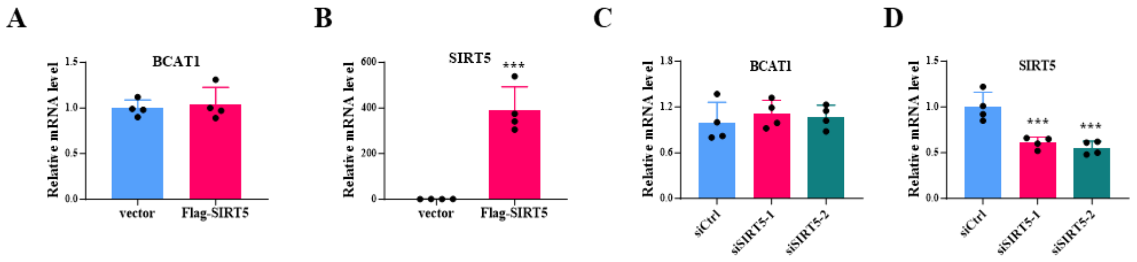

Supplemental Figure 5. Impact of SIRT5 on the mRNA levels of BCAT1. (A-B) qPCR analysis of SIRT5 and BCAT1 mRNA levels in U251 cells transfected with vector or SIRT5-Flag plasmid. (C-D) qPCR analysis of SIRT5 and BCAT1 mRNA levels in U251 cells transfected with scramble siRNA or SIRT5 siRNA. Data are presented as mean ± SD (n=4). ****p*<0.001.

Supplemental Figure 6

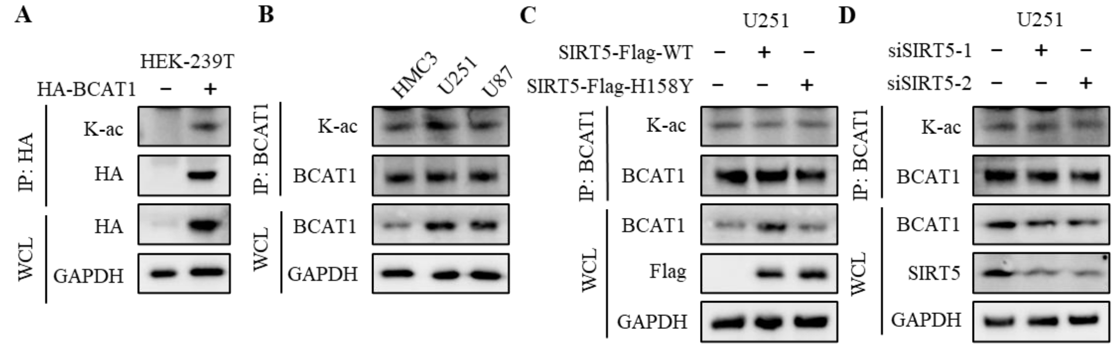

Supplemental Figure 6. Impact of SIRT5 on BCAT1 acetylation. (A) IP and western blot analysis of BCAT1 succinylation levels in HEK-293T cells transfected with HA-BCAT1 plasmid. (B) IP and western blot analysis of endogenous BCAT1 succinylation levels in HMC3, U87 and U251 cells. (C) IP and western blot analysis of BCAT1 succinylation levels in U251 cells transfected with vector or SIRT5-Flag plasmid. (D) IP and western blot analysis of BCAT1 succinylation levels in U251 cells transfected with scramble siRNA or SIRT5 siRNA.
