# Supplementary material for: SIRT5-mediated BCAT1 desuccinylation and stabilization leads to ferroptosis insensitivity and promotes cell proliferation in glioma
Source: Cell Death Dis. 2025 Apr 7;16(1):261. doi: 10.1038/s41419-025-07626-9 (PMC11977203; doi:10.1038/s41419-025-07626-9)
Supplement: Supplementary file 2 — Table S1 [file 41419_2025_7626_MOESM2_ESM.docx]

**Supplemental Table 1**. **Primers used in this study**

| **Construction of recombinant plasmid** | |
| --- | --- |
| SIRT5-F | 5’-AAATCTAGAGCTAGCGAATTCATGCGACCTCTCCAGATTGTC-3’ |
| SIRT5-R | 5’-ATCCGATTTAAATTCGAATTCTTAAGAAACAGTTTCATTTTCATGA-3’ |
| SIRT5-H158Y-F | 5’-AACCTTCTGGAGATCTATGGTAGCTTATTTAAA-3’ |
| SIRT5-H158Y-R | 5’-TTTAAATAAGCTACCATAGATCTCCAGAAGGTT-3’ |
| BCAT1-pLV-F | 5’-TATAGGGCCCGGGTTGGATCCGCCACCATGAAGGATTGCAGTAACGG-3’ |
| BCAT1-pLV-R | 5’-AGGCCGGAGACGCGTGGATCCTCAGGATAGCACAATTGTCC-3’ |
| BCAT1-F | 5’-ACCGAGATCTCTCGAGGTACCATGAAGGATTGCAGTAACGG-3’ |
| BCAT1-R | 5’-GATCCCCGCGGCCGCGGTACCTCAGGATAGCACAATTGTCC-3’ |
| BCAT1-K26R-F | 5’-GTGGTGGGGACTTTTAGGGCTAGAGACCTAATA-3’ |
| BCAT1-K26R-R | 5’-TATTAGGTCTCTAGCCCTAAAAGTCCCCACCAC-3’ |
| BCAT1-K28R-F | 5’-GGGACTTTTAAGGCTCGAGACCTAATAGTCACA-3’ |
| BCAT1-K28R-R | 5’-TGTGACTATTAGGTCTCGAGCCTTAAAAGTCCC-3’ |
| BCAT1-K39R-F | 5’-CCAGCTACCATTTTAAGGGAAAAACCAGACCCC-3’ |
| BCAT1-K39R-R | 5’-GGGGTCTGGTTTTTCCCTTAAAATGGTAGCTGG-3’ |
| BCAT1-K176R-F | 5’-CCTTCTCTTGGAGTCAGGAAGCCTACCAAAGCC-3’ |
| BCAT1-K176R-R | 5’-GGCTTTGGTAGGCTTCCTGACTCCAAGAGAAGG-3’ |
| BCAT1-K305R-F | 5’-CAGTGGGGTGAATTTAGGGTGTCAGAGAGATAC-3’ |
| BCAT1-K305R-R | 5’-GTATCTCTCTGACACCCTAAATTCACCCCACTG-3’ |
| **Quantitative RT-PCR** | |
| BCAT1-qPCR-F | 5’-CGGAGAAGGAGGATCAAAAGAGGT-3’ |
| BCAT1-qPCR-R | 5’-ATGAGCCAGGGTGCAATGACAGGT-3’ |
| SIRT5-qPCR-F | 5’-TGGAGGAGGTTGACAGAGAGCT-3’ |
| SIRT5-qPCR-R | 5’-ATTTTCATGACAGGCAAGGGCT-3’ |
| GAPDH-qPCR-F | 5’-ACAACTTTGGTATCGTGGAAGG-3’ |
| GAPDH-qPCR-R | 5’-GCCATCACGCCACAGTTTC-3’ |
