# Supplementary material for: SIRT5-mediated BCAT1 desuccinylation and stabilization leads to ferroptosis insensitivity and promotes cell proliferation in glioma
Source: Cell Death Dis. 2025 Apr 7;16(1):261. doi: 10.1038/s41419-025-07626-9 (PMC11977203; doi:10.1038/s41419-025-07626-9)
Supplement: Supplementary file 3 — Table S2 [file 41419_2025_7626_MOESM3_ESM.docx]

**Supplemental Table 2**. **BCAT1 succinylation sites were predicted using LMSuccSite platform.**

| \| Position \| Site residue \| Probability \| Prediction \| \| --- \| --- \| --- \| --- \| \| 2 \| K \| 0.013364 \| 0 \| \| 19 \| K \| 0.0853 \| 0 \| \| 26 \| K \| 0.836544 \| 1 \| \| 28 \| K \| 0.78366 \| 1 \| \| 39 \| K \| 0.649357 \| 1 \| \| 41 \| K \| 0.041515 \| 0 \| \| 70 \| K \| 0.007506 \| 0 \| \| 74 \| K \| 0.009323 \| 0 \| \| 99 \| K \| 0.149757 \| 0 \| \| 107 \| K \| 0.258169 \| 0 \| \| 134 \| K \| 0.072695 \| 0 \| \| 146 \| K \| 0.008412 \| 0 \| \| 176 \| K \| 0.692906 \| 1 \| \| 177 \| K \| 0.00965 \| 0 \| \| 180 \| K \| 0.002603 \| 0 \| \| 209 \| K \| 0.068686 \| 0 \| \| 215 \| K \| 0.47002 \| 0 \| \| 222 \| K \| 0.028421 \| 0 \| \| 305 \| K \| 0.583893 \| 1 \| \| 346 \| K \| 0.160147 \| 0 \| \| 360 \| K \| 0.20128 \| 0 \| |
| --- | --- | --- | --- | --- | --- | --- | --- | --- | --- | --- | --- | --- | --- | --- | --- | --- | --- | --- | --- | --- | --- | --- | --- | --- | --- | --- | --- | --- | --- | --- | --- | --- | --- | --- | --- | --- | --- | --- | --- | --- | --- | --- | --- | --- | --- | --- | --- | --- | --- | --- | --- | --- | --- | --- | --- | --- | --- | --- | --- | --- | --- | --- | --- | --- | --- | --- | --- | --- | --- | --- | --- | --- | --- | --- | --- | --- | --- | --- | --- | --- | --- | --- | --- | --- | --- | --- | --- | --- |
