# Supplementary material for: SIRT5-mediated BCAT1 desuccinylation and stabilization leads to ferroptosis insensitivity and promotes cell proliferation in glioma
Source: Cell Death Dis. 2025 Apr 7;16(1):261. doi: 10.1038/s41419-025-07626-9 (PMC11977203; doi:10.1038/s41419-025-07626-9)

Figure 1

B

SIRT5

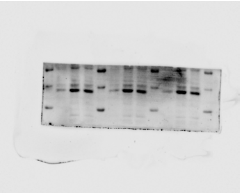

GAPDH

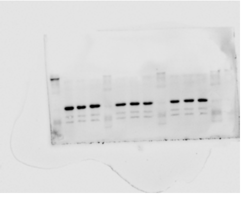

C

SIRT5

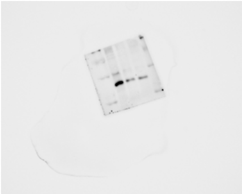

GAPDH

D

SIRT5

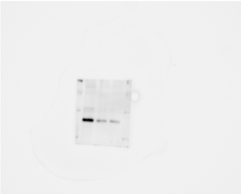

GAPDH

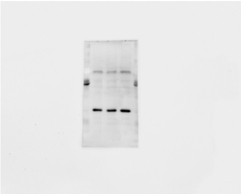

G

Flag

GAPDH

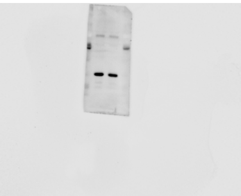

H

Flag

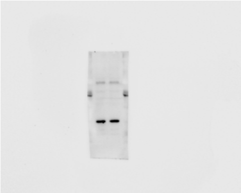

GAPDH

Figure 2

K

GPX4

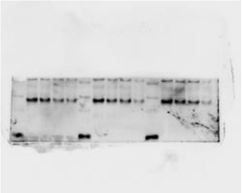

SLC7A11

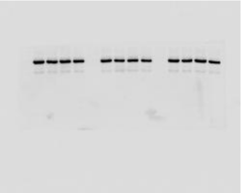

FTH1

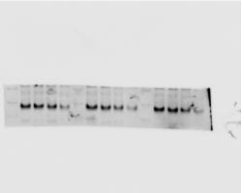

GAPDH

Figure 4

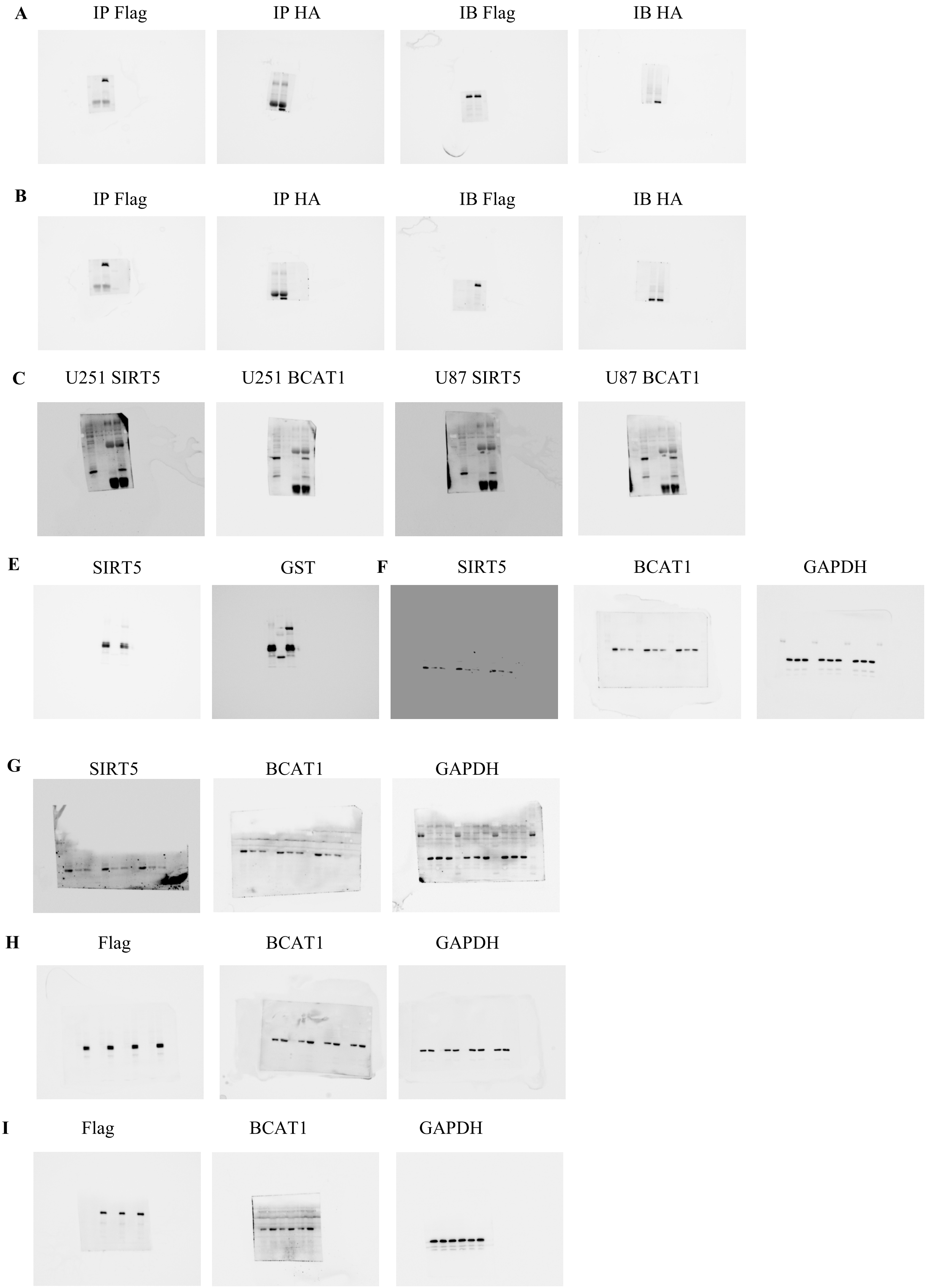
A
IP Flag
IP HA
IB Flag
IB HA
B
IP Flag
IP HA
IB Flag
IB HA
C
U251 SIRT5
U251 BCAT1
U87 SIRT5
U87 BCAT1
E
SIRT5
GST
F
SIRT5
BCAT1
GAPDH
G
SIRT5
BCAT1
GAPDH
H
Flag
BCAT1
GAPDH
I
Flag
BCAT1
GAPDH

Figure 4

J

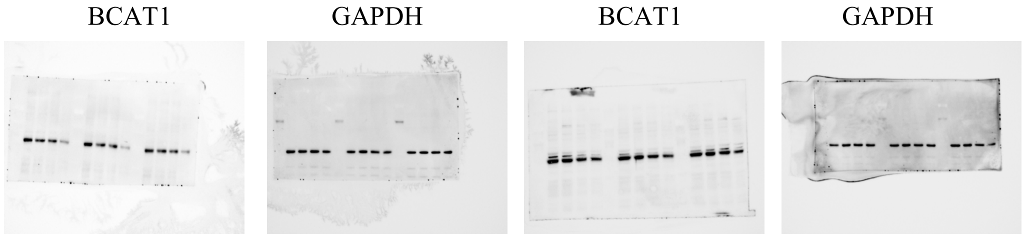
BCAT1
GAPDH
BCAT1
GAPDH

K

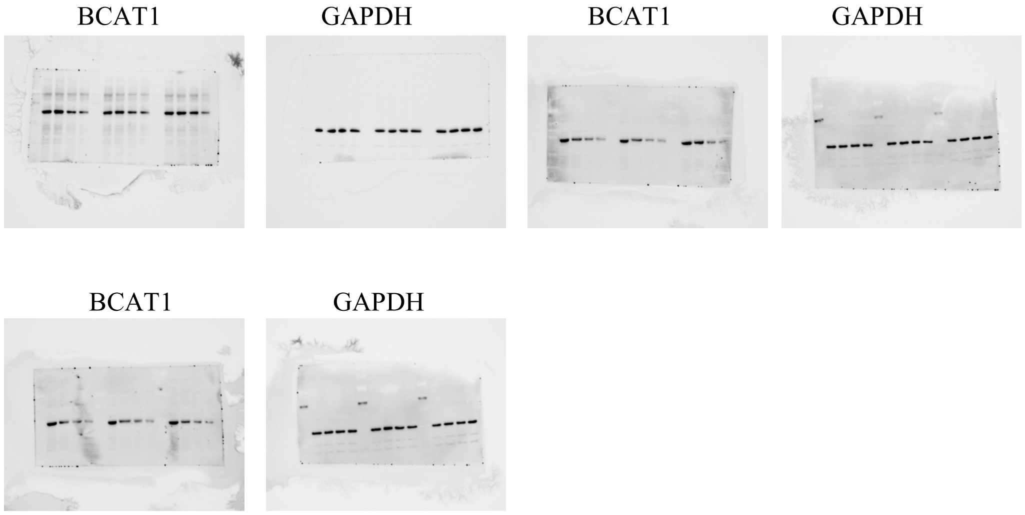
BCAT1
GAPDH
BCAT1
GAPDH
BCAT1
GAPDH

Figure 5

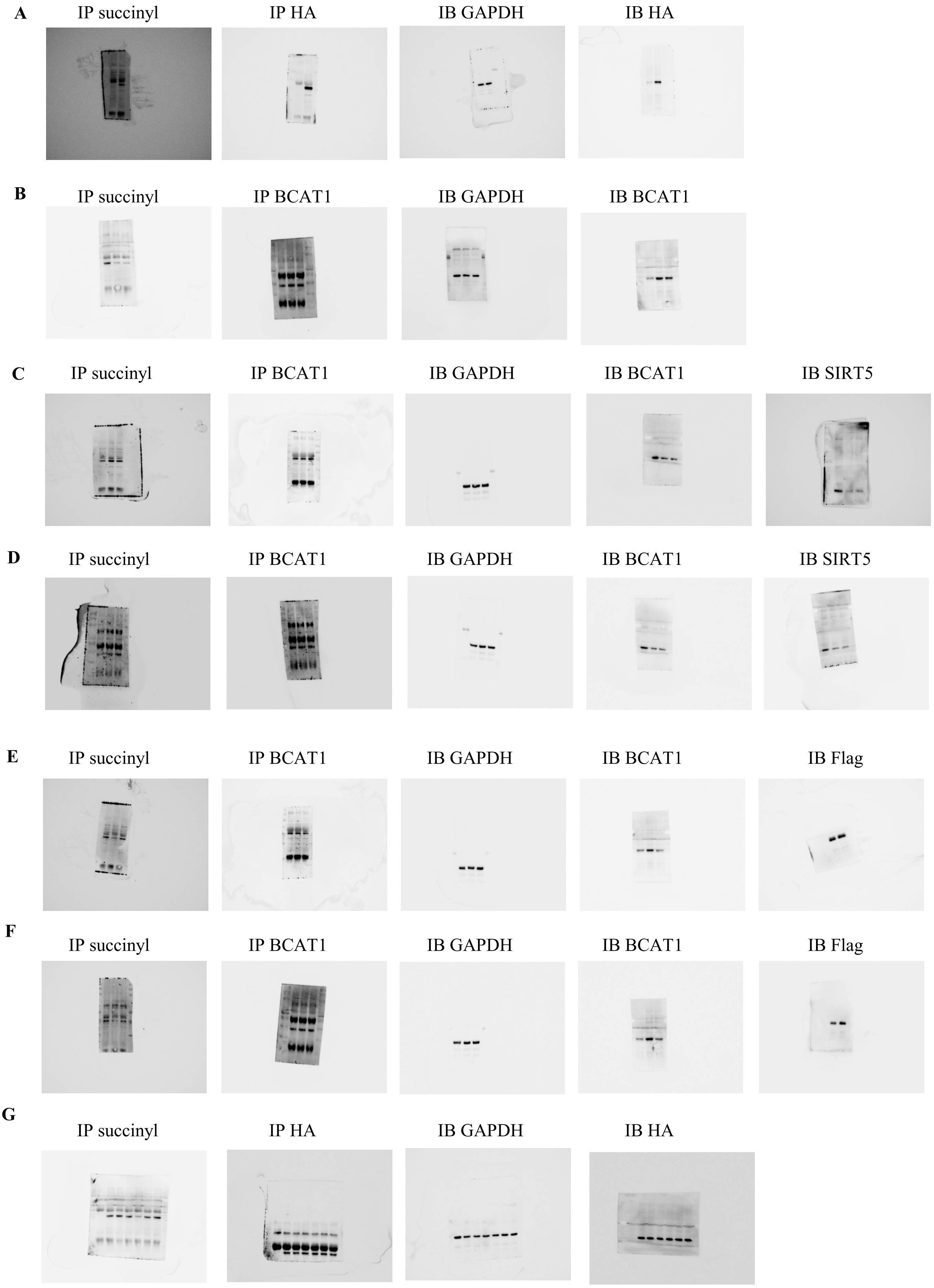
A
IP succinyl
IP HA
IB GAPDH
IB HA
B
IP succinyl
IP BCAT1
IB GAPDH
IB BCAT1
C
IP succinyl
IP BCAT1
IB GAPDH
IB BCAT1
IB SIRT5
D
IP succinyl
IP BCAT1
IB GAPDH
IB BCAT1
IB SIRT5
E
IP succinyl
IP BCAT1
IB GAPDH
IB BCAT1
IB Flag
F
IP succinyl
IP BCAT1
IB GAPDH
IB BCAT1
IB Flag
G
IP succinyl
IP HA
IB GAPDH
IB HA

Figure 5

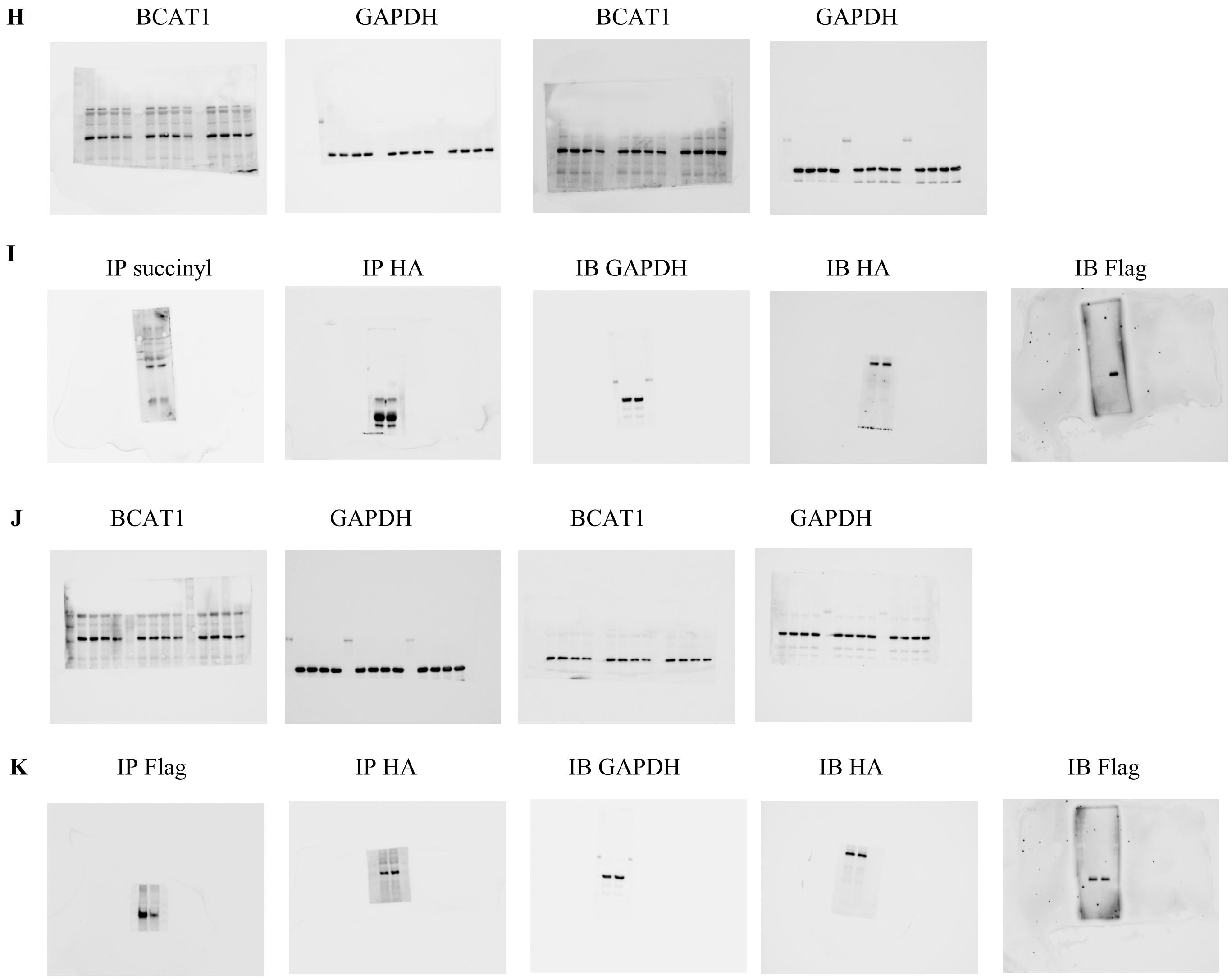
H
BCAT1
GAPDH
BCAT1
GAPDH
I
IP succinyl
IP HA
IB GAPDH
IB HA
IB Flag
J
BCAT1
GAPDH
BCAT1
GAPDH
K
IP Flag
IP HA
IB GAPDH
IB HA
IB Flag

Figure 6

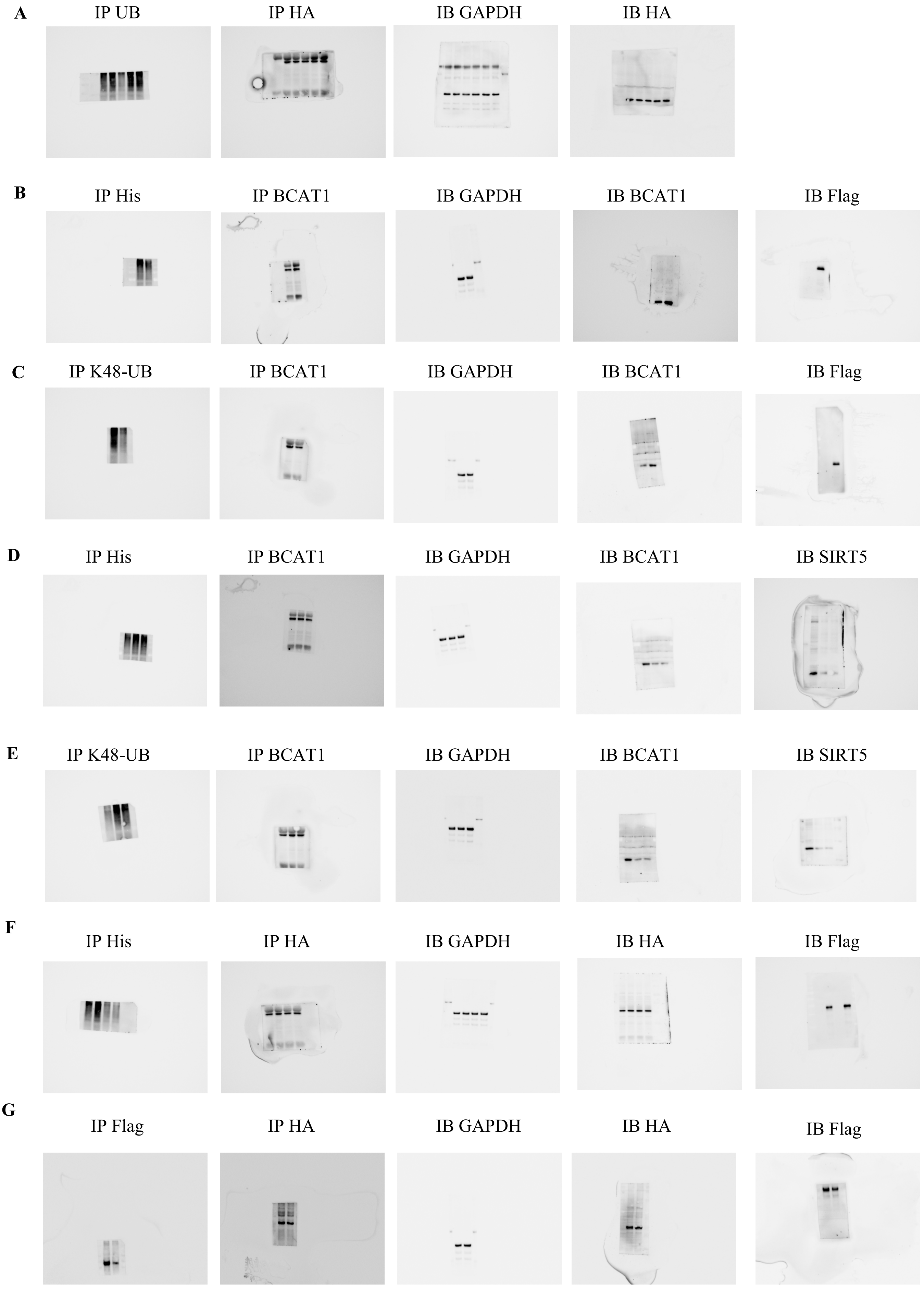
A
IP UB
IP HA
IB GAPDH
IB HA
B
IP His
IP BCAT1
IB GAPDH
IB BCAT1
IB Flag
C
IP K48-UB
IP BCAT1
IB GAPDH
IB BCAT1
IB Flag
D
IP His
IP BCAT1
IB GAPDH
IB BCAT1
IB SIRT5
E
IP K48-UB
IP BCAT1
IB GAPDH
IB BCAT1
IB SIRT5
F
IP His
IP HA
IB GAPDH
IB HA
IB Flag
G
IP Flag
IP HA
IB GAPDH
IB HA
IB Flag

Figure 6

H

IP CHIP

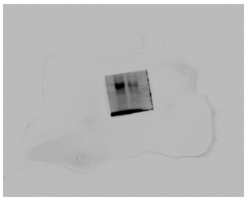

IP HA

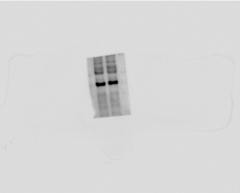

IB GAPDH

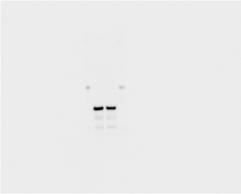

IB HA

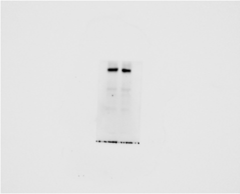

IB Flag

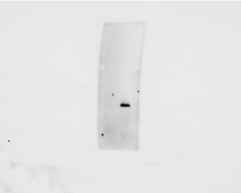

IB CHIP

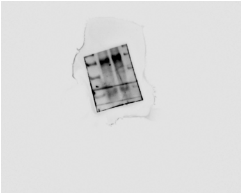

I

HA

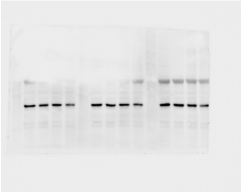

GAPDH

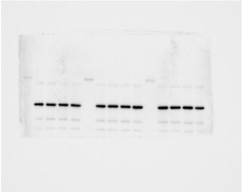

HA

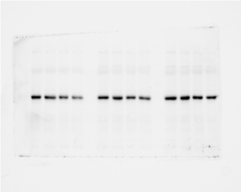

GAPDH

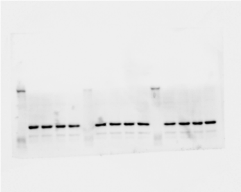

Figure 7

A

BCAT1

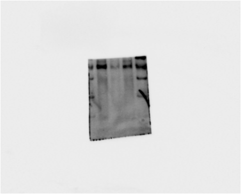

SIRT5

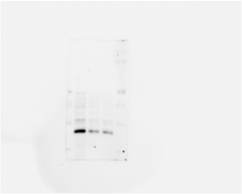

GAPDH

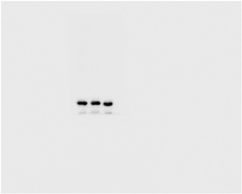

Figure 8

H

GPX4 SLC7A11 FTH1 GAPDH

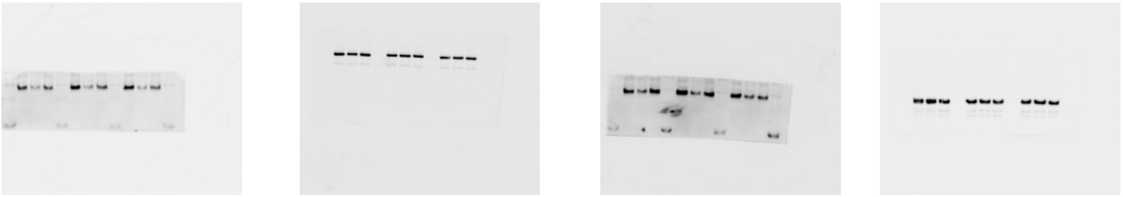

Supplement: Supplementary file 4 — Raw WB Data [file 41419_2025_7626_MOESM4_ESM.pdf]
